# WASp controls oriented migration of endothelial cells to achieve functional vascular patterning

**DOI:** 10.1242/dev.200195

**Published:** 2022-02-01

**Authors:** André Rosa, Wolfgang Giese, Katja Meier, Silvanus Alt, Alexandra Klaus-Bergmann, Lowell T. Edgar, Eireen Bartels-Klein, Russell T. Collins, Anna Szymborska, Baptiste Coxam, Miguel O. Bernabeu, Holger Gerhardt

**Affiliations:** 1 Integrative Vascular Biology Laboratory, Max Delbrück Center for Molecular Medicine in the Helmholtz Association (MDC), Berlin 13125, Germany; 2 DZHK (German Center for Cardiovascular Research), Berlin 10785, Germany; 3 Centre for Medical Informatics, Usher Institute, The University of Edinburgh, Edinburgh EH16 4UX, UK; 4 The Bayes Centre, The University of Edinburgh, Edinburgh EH8 9BT, UK; 5 Berlin Institute of Health (BIH), Berlin 10178, Germany

**Keywords:** Migration, Proliferation, WASp, F-actin, Vessel remodelling, Shear stress, Zebrafish, Human

## Abstract

Endothelial cell migration and proliferation are essential for the establishment of a hierarchical organization of blood vessels and optimal distribution of blood. However, how these cellular processes are quantitatively coordinated to drive vascular network morphogenesis remains unknown. Here, using the zebrafish vasculature as a model system, we demonstrate that the balanced distribution of endothelial cells, as well as the resulting regularity of vessel calibre, is a result of cell migration from veins towards arteries and cell proliferation in veins. We identify the Wiskott-Aldrich Syndrome protein (WASp) as an important molecular regulator of this process and show that loss of coordinated migration from veins to arteries upon *wasb* depletion results in aberrant vessel morphology and the formation of persistent arteriovenous shunts. We demonstrate that WASp achieves its function through the coordination of junctional actin assembly and PECAM1 recruitment and provide evidence that this is conserved in humans. Overall, we demonstrate that functional vascular patterning in the zebrafish trunk is established through differential cell migration regulated by junctional actin, and that interruption of differential migration may represent a pathomechanism in vascular malformations.

## Abstract (precis)

**Summary:** Regular diameter of developing veins and arteries in the zebrafish trunk is controlled by differential endothelial cell proliferation and WASp-driven directed cell migration.

## INTRODUCTION

The formation of a functional network of blood vessels with optimal hierarchy and morphology is a crucial process during development. Most blood vessels are derived from angiogenesis, a process that relies on the coordination of several endothelial cell (EC) behaviours including migration, proliferation and lumen formation (Eilken and Adams, 2010; Herbert and Stainier, 2011; Potente et al., 2011). After initial establishment of sprout connections (Betz et al., 2016), remodelling of vessels is accomplished by directed migration and proliferation within established vessels, in response to local tissue signalling (Wacker and Gerhardt, 2011) as well as haemodynamic forces (Chen et al., 2012; Franco et al., 2015; Kochhan et al., 2013; Udan et al., 2013).

EC migration (Michaelis, 2014), as well as signals that potentiate cell proliferation (Norton and Popel, 2016; Santos-Oliveira et al., 2015), have been extensively studied in different *in vivo* and *in vitro* systems. However, most of these studies investigated details of the initial sprouting process during the formation of the primitive vascular plexus. A detailed, quantitative understanding of the endothelial dynamics during vascular remodelling is lacking. Such understanding is required for the development of mathematical models that predict cellular behaviours and molecular mechanisms that control vascular remodelling and hierarchical patterning (Edgar et al., 2021).

Many aspects of cell migration and proliferation are known to rely on the control of the actin cytoskeleton (Heng and Koh, 2010; Svitkina, 2018). In the context of angiogenesis, several studies have shown that inhibition of F-actin polymerization compromises the process of elongation and rearrangement of cells (Angulo-Urarte et al., 2018; Sauteur et al., 2014) and the formation of cellular projections, such as filopodia, that instruct cell movements (Phng et al., 2013). Nevertheless, as many of these studies took advantage of powerful toxins that destabilize actin polymerization or interfere with acto-myosin contractility, we lack deeper insight into the molecular mechanism required for F-actin regulation during vascular remodelling. Specifically, the exact roles of actin regulators, and in particular of nucleators and nucleation promoting factors (NPF), in this process are not well understood.

Here, using the trunk vasculature of the zebrafish as a model system, we performed a detailed quantitative analysis of EC dynamics throughout the sprouting and remodelling phases. Our analysis showed that the exchange of cells between vessels and local proliferation determines the diameter of individual vessel segments. We quantitatively demonstrated that the balance of these processes is modulated by blood flow. We identified the actin NPF Wasb, previously thought to be selectively expressed in cells of haematopoietic origin, as a crucial regulator of this behaviour, acting partially by establishing junction-associated actin structures relevant for cell intercalation (Franco et al., 2016). Finally, we showed that loss of Wasb function *in vivo* drives vascular malformations, suggesting that interfering with directional migration may contribute to vascular pathologies.

## RESULTS

**Fig. 1.**
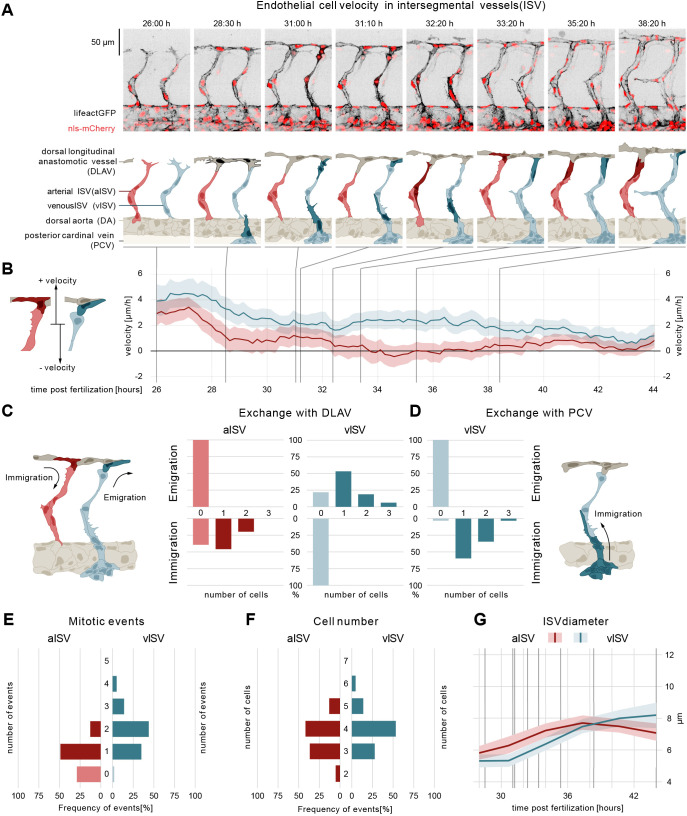
**ECs of arterial and venous ISVs show different velocity profiles.** (A) Montage of two ISV sprouts from 26-38 hpf. Top panel: live imaging of Tg[fliep:nls-mCherry; fliep:lifeactGFP]. Black, F-actin; red, nuclei. Bottom panel: pictogram of live imaging. Red ECs represent cells in aISV; blue ECs represent cells in vISV. Dark red ECs indicate cell division events (time point 32:20) and immigration of cells from DLAV to aISV (time points 33:20, 35:20-38:20). Dark blue cells indicate secondary sprouts (time point 28:30), vascular remodelling (time point 31:00, most ventral EC), cell division events (time point 31:00, most dorsal ECs), emigration of ECs towards the DLAV (time point 31:10, 33:20-38:20) and immigration of ECs from the PCV (time point 31:00-32:20). Dark grey cells represent cell anastomosis (time point 28:30). (B) EC velocity (µm/h) over time (hpf). Red, velocity for ECs in aISV; blue, velocity of ECs in vISV. Left pictogram demonstrates positive and negative velocity relative to dorsal and ventral movement, respectively. (C) Number of cells that exchange from ISVs with DLAV and fractions of ISVs that are affected. (D) Number of cells that exchange from PCV with vISV and corresponding fractions of vISVs. (E) Number of mitotic events in ISVs. (F) Cell number in ISVs 44 hpf and distribution among ISVs. (G) Vessel diameter (µm) over time (hpf) for aISV (red) and vISV (blue). Black vertical lines represent the equivalent time points in montage. Data are mean and 95% confidence interval.

In order to study the cellular and molecular processes driving vessel morphogenesis in the zebrafish trunk, we performed time-lapse imaging of embryos of endothelial-specific reporter lines with nuclear and filamentous actin labels (*fliep:nls-mCherry; fliep:lifeactGFP*) (Heckel et al., 2015; Phng et al., 2013) between 26 and 44 h post-fertilization (hpf) and systematically analyzed the trajectories of all endothelial cells in a ten-somite region (Fig. S1A). In short, at 26 hpf arterial sprouts [i.e. emerging from the dorsal aorta (DA)] comprise on average three ECs migrating dorsally (Fig. 1A). At 28-30 hpf the tip cells of neighbouring sprouts anastomose to form the dorsal longitudinal anastomotic vessel (DLAV) (Fig. 1A). At 31 hpf, venous sprouts emerge from the posterior cardinal vein (PCV), connecting to the existing arterial intersegmental vessels (ISVs). The remodelling of these connections between vessels (Geudens et al., 2019) ultimately leads to the formation of a balanced network of arterial and venous ISVs (aISVs and vISVs) by 44 hpf (Movie 1).

We tracked the cell nuclei, using them as an approximation for cell migration, and defined the direction of migration as positive or negative for dorsal (away from DA) or ventral (towards DA) movements, respectively (Fig. 1B). To distinguish the cells in the ISVs that will remodel into vISVs (future vISVs) from those in aISVs (future aISVs), we assessed the fate of each ISV in the remodelled network at 44 hpf. After rewinding the time-lapse sequence back to 26 hpf we marked each ISV as arterial or venous according to their final identity. In the following text, we will therefore refer to vISV and aISV even when describing developmental stages at which this identity is not yet visibly established through the final remodelling of the connections.

### Early EC migration patterns correlate with future vessel specification

Our quantitative analysis of EC migration revealed that cells residing in the future arteries and future veins are characterized by distinct velocities and directionality long before formal arterio-venous identity is established through remodelling (Fig. 1B; Fig. S1B). During sprouting, mean cell velocity in vISVs reached a maximum of 5 µm/h, compared with only 3.9 µm/h in aISVs. During anastomosis, dorsal mean cell velocity decreased to 2 µm/h and 1 µm/h in vISVs and aISVs, respectively. Subsequently, at the onset of the remodelling phase, both the speed and the direction of movement began to differ between vISVs and aISVs. In the sprouts that remained arterial, EC velocity continued to decrease and reversed direction, averaging at −0.6 µm/h. In contrast, in the sprouts that remodelled into veins, the orientation of movement remained the same and the velocity increased again to 3 µm/h. Finally, by 40 hpf, when the arterial-venous specification was complete, the velocity dropped to near zero in all ISVs.

### EC proliferation and directional migration differentially contribute to a balanced cell number in future arteries and veins

To understand the role of differential EC movement in ISV morphogenesis we followed the exchange of cells between vessel segments after the formation of the DLAV by measuring how many ECs entered (immigrated) or exited (emigrated) the ISVs (Fig. 1C, schematic). Remarkably, we found that arteries and veins ended up with similar cell numbers (three to four cells, Fig. 1F), but through different processes.

After DLAV formation, ECs in all ISVs no longer moved to and from the DA. The aISVs gained cells exclusively from the DLAV, with one or two cells immigrating from the DLAV to 60% of the observed aISVs (Fig. 1C, exchange with DLAV). No cells left the aISVs to contribute to the DLAV. In contrast, 76% of vISVs contributed one or two cells to the DLAV through emigration (Fig. 1C, exchange with DLAV). No cells left the DLAV to contribute to the vISVs. The other point of connection for the vISVs, the PCV, contributed with one or two cells in 92% of vISVs (Fig. 1D, exchange with PCV). Thus, the number of ECs in aISVs increased by immigration of cells from the DLAV, whereas vISVs received as many new cells from the PCV as they lose to the DLAV. Therefore, the developmental increase of ECs in vISVs needs to be achieved through a different process.

To address this, we quantified the number of cell divisions per vessel using the LifeAct-GFP marker, where mitosis can be recognized by cell rounding and increase of cortical F-actin (Fig. 1A; Movie 1) (Phng et al., 2013). In aISVs, a single cell division occurs in 50% of aISVs and two or more in 10% of aISVs (Fig. 1E). In vISVs, the rate of proliferation is higher, with one mitotic event occurring in 35% of vISVs and two mitotic events or more in 63% of vISVs (Fig. 1E). These results show that aISVs increase their EC number through combined cell immigration from the DLAV, and proliferation. In contrast, cell number in vISVs increases through cell proliferation, and the immigration and emigration of cells is evenly balanced.

These distinct morphogenic processes are accompanied by a steady and linear increase in vessel diameter as development progresses (Fig. 1G, diameter after remodelling: vISVs ∼8 µm; aISVs ∼7 µm).

### Vessel diameter control in the zebrafish trunk requires the actin regulator Wasb

**Fig. 2.**
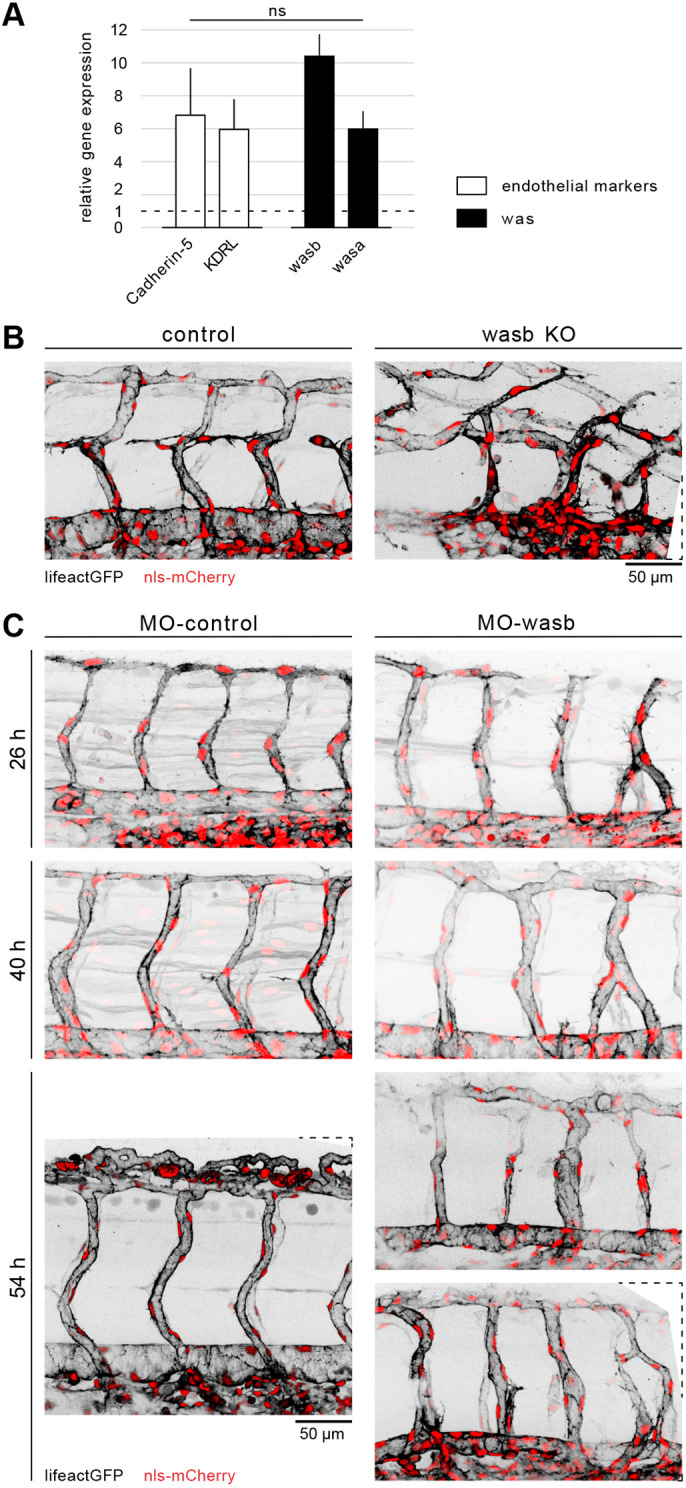
**Vascular morphology is dependent on Wasb.** (A) RT-PCR of 24 hpf FACS-processed ECs from Tg[fliep:eGFP]. Data are mean+s.e.m. (B) Trunk vasculature of 44 hpf Tg[fliep:nls-mCherry; fliep:lifeactGFP] F0 embryo injected with Cas9 mRNA (control) and Cas9 mRNA plus guide mRNA (wasb KO). (C) *MO-control* and *MO-wasb* Tg[fliep:nls-mCherry; fliep:lifeactGFP] embryos. Top panel of *MO-wasb* shows large vISVs with large diameter and aISVs with reduced diameter. Lower panel shows an example of a vascular malformation.

To identify molecular mechanisms responsible for the differential cell migration, we used fluorescence-activated cell sorting (FACS) on zebrafish ECs and assessed them for the expression of 40 known actin regulators using qPCR. We found that the *wasb* gene showed high levels of expression in ECs compared with non-endothelial cells at 24- and 48-hpf (Fig. 2A). The human homologue of Wasb, the Wiskott-Aldrich Syndrome protein (WASp, also known as WAS), was first isolated in lymphocytes (Derry et al., 1994) and identified as an important regulator of the actin cytoskeleton of myeloid and lymphoid cells (Thrasher and Burns, 2010). However, endothelial expression or function of WASp has not been reported. To investigate a potential role of *wasb* in vascular remodelling, we knocked-out *wasb* with CRISPR-Cas9 (Fig. 2B; Fig. S2A-B″). In F0 mutant embryos (*n*=45), the trunk vasculature showed a spectrum of vascular phenotypes, with severity increasing with the ratio of InDels (Fig. 2B; Fig. S2B′,B″). Embryos with a ratio of deletion of 100% (Fig. 2A) showed gross abnormalities, with a bent aorta and irregular diameter. Furthermore, the stereotypical distribution of ISVs was lost and instead several aberrant vessel connections were formed. Nonetheless, perfusion could still be observed (Movie 2). This phenotype was still observed, but less severe, in embryos with an 80% InDel frequency. Embryos with 40% InDels showed only enlarged vISVs and narrow aISVs compared with wild-type vessels (Fig. S2B′,B″). To further investigate the role of *wasb* in zebrafish vascular development we made use of previously validated *wasb* antisense morpholinos (Cvejic et al., 2008; Jones et al., 2013). Embryos injected with 8 ng of *wasb* morpholino (*MO-wasb*) developed aberrant ISV connections (three-way connections that did not resolve as well as direct ISV-ISV connections; Fig. 2C; Fig. S2C,C′) that persisted for up to 5 days post-fertilization (Movie 3). Furthermore, 54% of *MO-wasb* embryos showed ISVs with abnormal diameter (Fig. 2C), before vessel patterning was completely lost, resembling F0 crispant embryos with 40% frequency of InDels (Fig. S2B′). Increasing the dosage of morpholino to 16 ng (Fig. S2D) resulted in a phenotype that mostly resembled F0 crispant embryos with 100% and 80% frequency of InDels (Fig. 2B; Fig. S2D). The MO phenotype was fully rescued by endothelial-specific expression of Wasb-mCherry (FliGal4::UAS-wasb-mCherry) (Fig. S2E), demonstrating the specificity of *wasb* morpholinos and that endothelial *wasb* expression is necessary to establish a uniform vascular pattering of vISVs and aISVs.

### Wasb controls endothelial cell migration

**Fig. 3.**
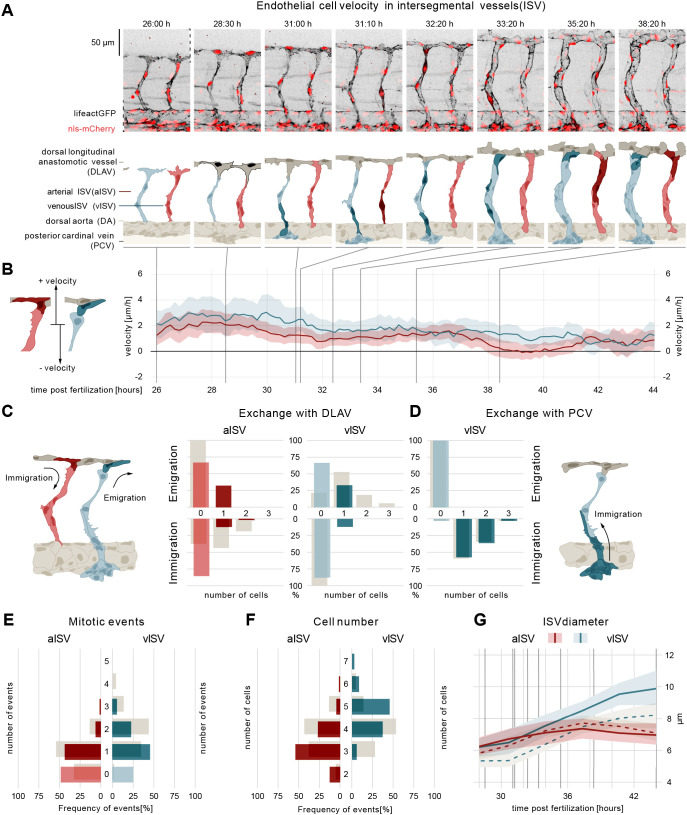
**Wasb is required for oriented cell migration.** (A) Montage of two ISV sprouts from 26 hpf to 38 hpf in *MO-wasb* embryo. Top panel: live imaging of Tg[fliep:nls-mCherry; fliep:lifeactGFP] *MO-wasb*. Black, F-actin; red, nuclei. Bottom panel: pictogram of live imaging. Red ECs represent cells in aISVs; blue ECs represent cells in vISVs. Dark red ECs indicate cell division events (time point 31:10) and emigration of cells from aISV to DLAV (time points 35:20-38:20). Dark blue and dark red cells indicate vascular remodelling (time point 31:00), cell division (time point 32:20, 33:20) and emigration of ECs towards the DLAV (time point 31:10, 33:20-38:20). Dark grey cells represent cell anastomosis (time point 28:30). (B) EC velocity (µm/h) over time (hpf). Red, velocity for ECs in aISVs; blue, velocity of ECs in vISV. Left pictogram demonstrates positive and negative velocity relative to dorsal and ventral movement, respectively. (C) Number of cells that exchange from ISVs with DLAV and fractions of ISVs that are affected (immigration from DLAV to aISV AKS test, *P*<0.05; emigration from vISV to DLAV AKS test, *P*<0.05; *MO-control* versus *MO-wasb*). (D) Number of cells that exchange from PCV with vISV and corresponding fractions of vISVs. (E) Number of mitotic events in ISVs (*MO-control* versus *MO-wasb* vISV, AKS test *P*<0.05). (F) Cell number in ISVs 44 hpf and distribution among ISVs (*MO-control* versus *MO-wasb* vISV, AKS test *P*<0.05). Grey bars (C-F) are values of *MO-control*. (G) Vessel diameter (µm) over time (hpf) for *MO-control* aISV (dotted redline), *MO-wasb* aISV (full redline), *MO-control* vISV (dotted blue line) and *MO-wasb* vISV (full blue line). Black vertical lines represent the equivalent time points in montage. Data are mean and 95% confidence interval.

WASp belongs to a family of NPFs required for diverse cellular processes, including cell migration (Burns et al., 2001). Given that loss of *wasb* expression triggered vascular malformations and impaired vessel diameter control, we hypothesized that Wasb may also regulate EC migration. To test this, we performed quantitative morphodynamics analyses in embryos injected with 8 ng of *MO-wasb* (Fig. 3; Fig. S3A-C)*.* Similar to control embryos (Fig. 1A,B), ECs in arterial sprouts from *MO-wasb* moved with a positive velocity (Fig. 3A,B; Movie 4), but on average ∼50% slower compared with their *MO-control* injected siblings. Although DLAV formation and vessel remodelling proceeded in *MO-wasb* embryos, the characteristic reduction in velocity during DLAV formation was less prominent. Intriguingly, the differential velocity profile characterizing aISVs and vISVs during vessel remodelling was lost in *MO-wasb* embryos (Fig. S3D).

### *wasb* is required for balanced EC migration between vessels

To further investigate the cellular mechanisms that lead to the defective vascular patterning in the absence of *wasb*, we quantified EC exchange between the vessels in the trunk. This analysis revealed a profound defect in differential migratory behaviour between veins and arteries.

In *MO-wasb* embryos the immigration of cells from the DLAV into aISVs was substantially reduced to one cell in less than 20% of aISVs and two cells in only 2% of aISVs, compared with 40% and 20% in *MO-control*, respectively (Fig. 3C). Moreover, in *MO-wasb* embryos some ECs moved from the aISVs to the DLAV in 40% of the vessels. We never observed this type of event in the control embryos. At the same time, cell proliferation in the arteries was not significantly altered (Fig. 3D). Although the movement of cells from and into the aISVs resulted in slightly more aISVs that harboured only three ECs (Fig. 3F), the overall number of ECs in the aISVs was not statistically different in *MO-wasb* compared with *MO-control*, and the aISV diameter remained largely unaffected (Fig. 3G).

The altered cell movement patterns were even more pronounced in vISVs. We observed a strong reduction in the number of cells that emigrated from vISVs to DLAV compared with *MO-control* embryos (compare Fig. 1C with Fig. 3C). Moreover, although in *MO-control* embryos ECs never immigrate from the DLAV to the vISVs, in *MO-wasb* embryos this aberrant behaviour occurred for 10% of the vISVs (Fig. 3C). Although the contribution of cells from the PCV to vISVs was not affected (Fig. 3D), the number of mitotic events in *MO-wasb* vISVs was significantly reduced (Fig. 3E). Of note, the reduced proliferation was not sufficient to rebalance cell numbers. The net effect of the reduced emigration from and increased immigration to the vISVs, resulted in an overall increase in the number of cells per vessel in *MO-wasb* (Fig. 3F), which explained the increased vessel diameter (Fig. 3G). Overall, this quantitative analysis demonstrates that Wasb plays an important role in the coordination of directed movement of ECs during vessel remodelling.

### Reduced wall shear stress increases variability of vessel diameter

During development, ECs are subjected to wall shear stress (WSS) exerted by the flowing blood. The magnitude of this force is dependent on flow rate, blood viscosity and the physical dimensions of the blood vessels (Ballermann et al., 1998). Given the alterations in vessel diameters observed in *MO-wasb* embryos, we asked how this altered network would affect the WSS within ISVs. To address this, we constructed an *in silico* stereotypical ISV network with vessel diameter measurements from each zebrafish (Fig. S4A,B). These models were then used to compute blood flow rate, direction and WSS evolution within the remodelling aISVs and vISVs.

According to our simulations, in *MO-control* embryos the WSS in vISVs is initially higher than in aISVs (Fig. S4C). As the remodelling progresses, aISVs become vessels with higher WSS than vISVs. This mirrors the dynamic changes in vessel diameter (Fig. S4C and Fig. 1G). However, in *MO-wasb*, our simulation predicted that aISVs experience higher WSS at the beginning of the remodelling phase (Fig. S3C). Interestingly, although the WSS in aISVs steadily increased, the vessel diameter remained constant. Upon closer examination of the simulation, we found that this effect is the consequence of a global network effect driven by the enlargement of vISVs (Fig. S3C; Fig. 3G)

**Fig. 4.**
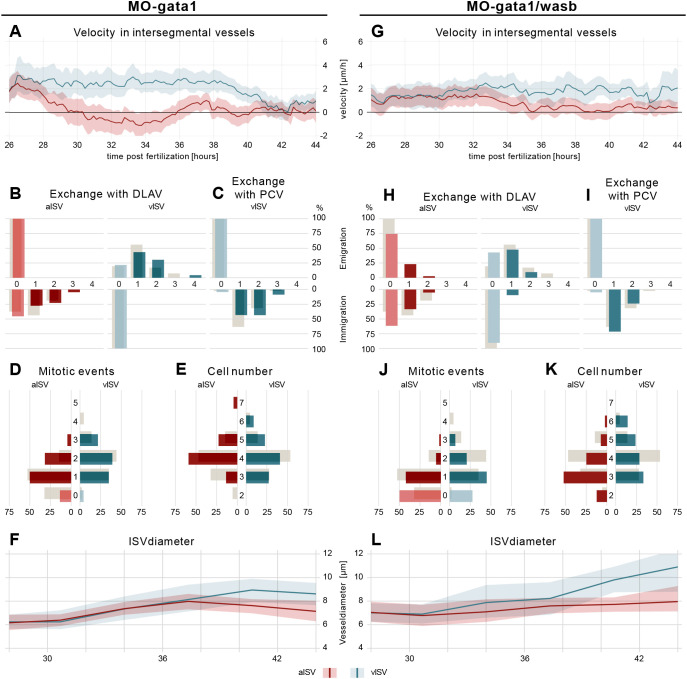
**WSS prevents vessel diameter and cell number variability.** (A) EC velocity (µm/h) over time (hpf) of *MO-gata1* embryos. Red, velocity for ECs in aISVs; blue, velocity of ECs in vISV. (B) Number of cells that exchange from ISVs with DLAV and fractions of ISVs that are affected (AKS test *P*>0.05, *MO-control* versus *MO-gata1*). (C) Number of cells that exchange from PCV with vISV and corresponding fractions of vISVs. (D) Number of mitotic events in ISVs (AKS test *P*>0.05, *MO-control* versus *MO-gata1*). (E) Cell number in ISV at 44 hpf and distribution among ISVs (AKS test *P*>0.05, *MO-control* versus *MO-gata1*). (F) Vessel diameter (µm) over time (hpf) for *MO-gata1* ISV (red line, aISV; blue line, vISV). (G) EC velocity (µm/h) over time (hpf) *of MO-gata1/wasb* embryos. Red, velocity for ECs in aISVs; blue, velocity of ECs in vISV. (H) Number of cells that exchange from ISVs with DLAV and fractions of ISVs that are affected (AKS test *P*>0.05, *MO-gata1* versus *MO-gata1/wasb*). (I) Number of cells that exchange from PCV with vISV and corresponding fractions of vISVs. (J) Number of mitotic events in ISVs (AKS test aISV *P*<0.05, *MO-gata1* versus *MO-gata1/wasb*; AKS test vISV *P*>0.05 *MO-gata1* versus *MO-gata1/wasb*). (K) Cell number in ISVs 44 hpf and distribution among ISVs (AKS test aISV *P*<0.05, *MO-gata1* versus *MO-gata1/wasb*; AKS test vISV *P*>0.05, *MO-gata1* versus *MO-gata1/wasb*). (L) Vessel diameter (µm) over time (hpf) for *MO-gata1/wasb* ISV (red line, aISV; blue line, vISV). Grey bars (B-E,H-K) are values of *MO-control*. Data are mean and 95% confidence interval.

As WSS is known to influence the behaviour of ECs, particularly EC migration (Franco et al., 2015, 2016), we asked whether the predicted WSS pattern alteration in *MO-wasb* embryos would serve to enhance or counteract the altered EC velocity and the final vessel morphology. To address this experimentally, we reduced WSS *in vivo* by the injection of morpholinos against *gata1* (also known as *gata1a*) (*MO-gata1*) preventing erythroid differentiation (Galloway et al., 2005). This approach reduced blood viscosity and thus WSS threefold by eliminating circulating erythrocytes (Lee et al., 2017). Morphodynamic analysis of *MO-gata1* embryos demonstrated that ECs in vISVs migrated consistently with an average of ∼3 µm/h from 26 hpf until 40 hpf, whereas in *MO-control* embryos EC velocity was altered during DLAV formation and vessel remodelling. In contrast, ECs in aISVs continuously decreased their velocity during that time, whereas in *MO-control* embryos velocity was gradually decreased (Fig. 4A; Fig. S5; Movie 5). In sum, these alterations in EC velocity established differential arterio-venous movement of cells that began earlier than in *MO-control* embryos. Although the decrease in blood viscosity resulted in a prolonged period of differential cell movement, overall this alteration did not have a significant impact on cell exchange or proliferation (Fig. 4B-D). However, we noticed an increased variability in cell number and vessel diameter (Fig. 4E,F), suggesting that WSS contributes to patterning robustness.

### Cross-correlation analysis reveals a balancing mechanism that controls vessel diameter

To gain insight on how cellular behaviour (cell division and cell exchange) influences the outcome of vessel diameter, we compared mitotic events and net immigration (net immigration=EC_immigration_−EC_emigration_) with final vessel diameter for each ISV (Fig. S6A-E). With these comparisons we observed that cell numbers in the ISVs with reduced proliferation rate are compensated by immigration of ECs from neighbouring segments and, as a result are able to establish the appropriate vessel diameter (Fig. S6A-A″,E). This balance is affected by reducing WSS in *MO-gata1* embryos, particularly in vISVs where vessel diameter solely correlates with net immigration (Fig. S6C-C″,E) and is controlled by *wasb* (Fig. S6B-B″,E). Loss of *wasb* results in the loss of any correlation between cell proliferation or net immigration with aISV diameter.

### Reduced WSS partially rescues migration defects in *MO-wasb*

Given that Wasb deficiency dramatically alters the balanced distribution of cells in the trunk vasculature, and that WSS appears to help balance immigration against proliferation, we investigated the impact of decreased WSS in the background of *MO-wasb* embryos by co-injecting morpholinos that target *gata1* and *wasb* (*MO-gata1/wasb*). Surprisingly, the morphodynamic analysis revealed a partial rescue of migration velocity patterns between aISVs and vISVs (Fig. 4G; Movie 6). The EC velocity of *MO-gata1/wasb* in vISVs is maintained at ∼2 µm/h and in aISVs decreased to 0 µm/h from 33 hpf to 44 hpf (Fig. 4G; Fig. S5E,F,I). This alteration is followed by an increased tendency [albeit not significant compared with *MO-wasb*, adapted Kolmogorov-Smirnov test (AKS) *P*>0.05] of EC to immigrate to aISVs and to emigrate from vISVs (Fig. 4H, EC exchange with the PCV is unaltered: Fig. 4I). Interestingly, the frequency of mitotic events (Fig. 4J) was not restored compared with *MO-wasb* (AKS *P*>0.05), suggesting that WSS primarily influences migratory behaviour. This hypothesis is supported by the observation that in aISVs, diameter increase (Fig. 4L; Fig. S5G,H) correlates with net immigration and in vISV diameter decrease negatively correlates with net immigration (Fig. S6D-D″,E). Thus, reduced WSS to a degree ameliorates *wasb*-dependent diameter abnormalities by partially rescuing differential cell movement. However, the natural balance of directed migration and proliferation is not restored, which would explain the increased variation of cell number and diameter (Fig. 4K,L).

### Wasb regulates endothelial actin organization

**Fig. 5.**
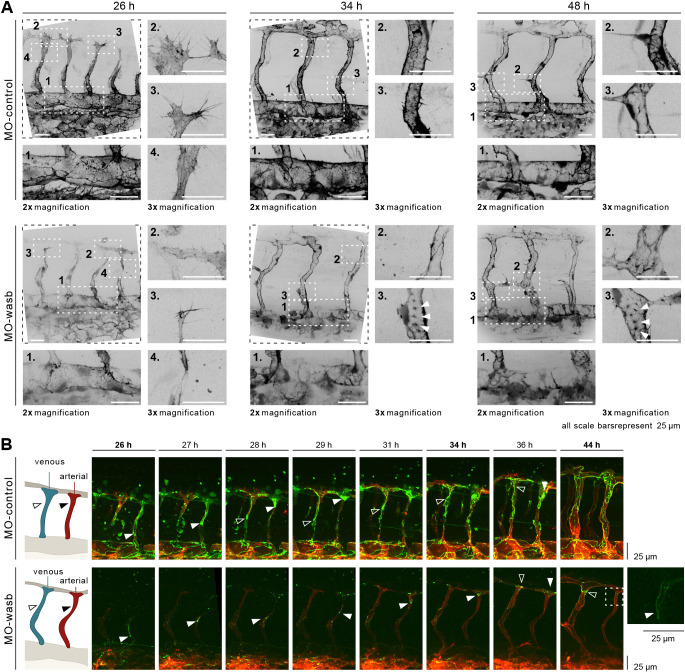
**Wasb is required for F-actin regulation and junctional Pecam1 localization.** (A) Upper panel: trunk vasculature of Tg[fliep:lifeactGFP] *MO-control*. F-actin is mostly prominent at 26 hpf in filopodia (inset 3), cell-cell contacts of anastomosis (inset 2), cell cortex and stress fibres (inset 4). In the DA, F-actin accumulates at cell junctions (inset 1). At 34 hpf and later at 48 hpf F-actin is enriched at junctions of ISV (insets 2 and 3) and DA (insets 1). Lower panel: trunk vasculature of Tg[fliep:lifeactGFP] *MO-wasb*. F-actin is decreased during anastomosis (inset 3) with heterogeneous accumulation at the cortex and loss of stress fibres (inset 4). Filopodia still show F-actin accumulation (inset 2). In the DA junctional F-actin is lost (inset 1). At 34 hpf and later at 48 hpf junctional accumulation is heterogenous (insets 2) and in the form of puncta (insets 3). In the DA F-actin has heterogeneous accumulation (insets 1). (B) *MO-control*: Pecam1 (green) junctional localization in remodelling vessels labels migratory cells. White arrowhead labels migratory stalk EC in aISV. Dorsal movement from 26 to 29 h. Diffuse Pecam1 localization at 34 hpf. Ventral movement at 36 hpf. Black arrowhead labels migratory stalk EC in vISV with dorsal movement from 28 to 36 hpf. *MO-wasb*: Pecam1 is lost or reduced in EC junctions. White arrowhead labels migratory stalk EC in aISVs with dorsal movement from 26 to 36 hpf. Inset (right) labels second stalk cell with ventral-to-dorsal orientation. Black arrowhead labels migratory EC entering vISV from 36 to 44 h. Red labels ECs membrane.

Polymerization of branched actin by the Arp2/3 complex is required for cell migration (Cooper and Pollard, 1985). Nucleation activity of Arp2/3 is regulated by NPF-like Wasb (Jones et al., 2013). To understand how the actin cytoskeleton is affected by decreased *wasb* expression, we imaged F-actin in ECs of Tg(*fliep:lifeactGFP*) embryos injected with *wasb* morpholino. We observed pronounced defects in the organization of junctional actin upon *wasb* knockdown. Although in the sprouting vessels, F-actin was not appreciably affected (Fig. 5A, inset 4; Fig. S7A), the junction-associated actin in the aorta was already decreased at 26 hpf and did not recover during the observation time (Fig. 5A, inset 1; Fig. S7A). During remodelling, in both venous and arterial ISVs, the amount of junctional F-actin was significantly decreased (Fig. 5A 48 hpf, inset 2-3; Fig. S7B) and we observed junction-associated actin puncta. We also observed that, in sprouting vessels of *MO-wasb* embryos, the filopodia and lamellipodia, although still present, showed heterogeneous distribution of F-actin that differed from the control embryos. These results suggest that the assembly of continuous actin filaments in ECs, in particular along cell junctions, is dependent on Wasb.

Sauter and colleagues proposed that EC elongation required for angiogenic sprouting is dependent on junctional reorganization of Cadherin 5 and actin polymerization (Sauteur et al., 2014). Similarly, we have reported that blocking of general actin polymerization with latrunculin B or knockdown of the endothelial actin nucleator Fmnl3 disrupts both cell elongation and endothelial cell rearrangement that jointly establish longitudinal parallel junctions within the ISVs (Phng et al., 2015). To investigate the impact of *wasb* knockdown on cell interface elongation and Cadherin 5 localization, we used the Cadherin labelling transgenic line *Tg*(*ve-cad:ve-cadTS*) (Lagendijk et al., 2017). Although VE-Cadherin-TS still localized to cell junctions in embryos injected with *wasb* morpholino (Fig. S7C), the most dorsal part of the ISVs lacked continuous junctional labelling (Fig. S7D), similar to the phenotype caused by latrunculin B, indicating that Wasb-dependent actin polymerization is required for junction elongation.

### Wasb is necessary for junctional localization of Pecam1 in ECs

Pecam1 (CD-31) is a junctional protein with important functions for junction integrity (Privratsky et al., 2011), cell migration (Cao et al., 2002; O'Brien et al., 2004; Zhu et al., 2010) and endothelial shear stress response (Tzima et al., 2005). This prompted us to investigate the distribution and dynamics of this protein during vessel remodelling and the context of junctional defects caused by the depletion of *wasb*.

In zebrafish embryos with endothelial-specific expression of Pecam1-EGFP (*Tg[fli1a:pecam1-EGFP]^ncv27^*) (Ando et al., 2016), Pecam1 clearly localized to the junctions in the aorta as reported previously (Fig. 5B). In sprouting and remodelling ISVs, Pecam1 showed mostly indistinct, perijunctional localization (Fig. 5B, time interval from 26-36 hpf), with the exception of the rear of actively migrating cells, where it formed a small dense cluster (Fig. 5B, white arrowheads). Intriguingly, we observed that when cells switched direction, Pecam1 localization temporarily became diffuse again and resumed its junctional localization shortly before a new direction was established (Fig. 5B, control panel time point 29-34 hpf, white arrowhead). Towards the end of the remodelling phase, Pecam1 in the ISVs acquired junctional localization resembling the patterns in the aorta (Fig. 5B, time 44 hpf).

In *MO-wasb*-injected embryos, the junctional accumulation of Pecam1 in the aorta, as well as in the ISVs (Fig. 5B, *MO-wasb*; Fig. S7E) was impaired, with only a few puncta accumulating at the rear of migratory cells (Fig. 5B, *MO-wasb*, white arrowhead). Interestingly, as *MO-wasb* cells in aISVs fail to reverse their migratory direction, their residual Pecam1 expression never became diffuse (Fig. 5B, time point 44 hpf, inset). To investigate whether the loss of junctional Pecam1 in the absence of Wasb is a direct consequence of reduced actin polymerization or of Wasb activity as a scaffold protein (Charras and Yap, 2018), we treated *Tg[fli1a:pecam1-EGFP]^ncv27^* embryos with a low dose of latrunculin B (Phng et al., 2013). The treatment induced a developmental delay and decreased EC migration, as previously reported. Importantly, junctional accumulation of Pecam1 in these embryos was lost (Fig. S7F), suggesting that Wasb-mediated actin nucleation is required for junctional accumulation of PECAM.

### WASp is required for oriented cell migration in human ECs

To determine whether endothelial functions of Wasb are conserved*,* we examined the role of human WASp in cultured human umbilical vein endothelial cells (HUVECs) using small interfering RNA against WASp (siWAS) (Fig. S8A,B).

**Fig. 6.**
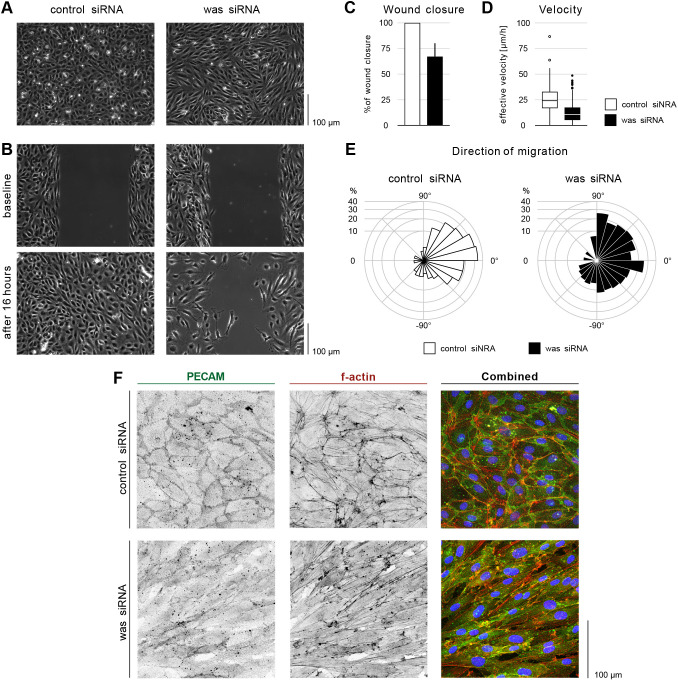
**WASp controls migration and PECAM1 localization in HUVECs.** (A) Phase contrast images of control and was siRNA-treated HUVECs in a confluent monolayer. (B) Scratch wound assay for control and was siRNA-treated HUVECs at baseline (immediately after removing barrier) and after16 h. (C) Quantification of wound closure at 16 h. Data are mean±s.d. of three independent experiments (six biological replicates). (D) Effective velocity of HUVECs during wound closure assay. Samples have a significant velocity difference, *P*<0.0001 (Welch's two sample *t*-test). (E) Rosette graph showing the prevalent cell direction during wound closure in control and was siRNA-treated cells. Comparison between graphs: *P*<0.001 (Watson's two-sample test of homogeneity). (F) HUVECs treated for control and was siRNA stained for f-actin (Phalloidin, red in combined), PECAM1 (green in combined) and nuclei (DAPI, blue).

In a confluent monolayer siWAS-treated cells changed morphology from a characteristic cobblestone to an elongated shape (Fig. 6A). Cells also formed streams and swirls, a phenotype reminiscent of the effect of depletion of Hippo pathway mediators, YAP and TAZ (Neto et al., 2018), caused by defects in junctional remodelling that prevent dynamic cell rearrangements. Based on these similarities, we hypothesized that WASp may be similarly required in ECs to facilitate shuffling between neighbours.

To examine the role of WASp in cell migration, we performed a wound closing assay (WCA; Jonkman et al., 2014) and time-lapse imaging (Fig. 6B). After 16 h from assay onset, siWAS-treated cells showed a 30% decrease in free-space coverage compared with control (siCTR) cells (Fig. 6C). This effect could be explained by either defects in cell migration, decreased proliferation or increased apoptosis. We assessed the number of proliferating cells by immunofluorescence staining against Ki67 (Scholzen and Gerdes, 2000). Although initially (first 6 h) siCTR cells proliferated faster than siWAS, this effect was not accompanied by faster wound closure during this time period (Fig. S8C,E). We also did not observe substantial difference in the number of apoptotic events in time-lapse recordings (Fig. S8F). After 16 h, the total number of cells in siWAS per mm^2^ of the monolayer was higher than in siCTR. All these observations support the interpretation that reduced wound closure in siWAS is due to impaired migration. Nuclei tracking in time-lapse recordings revealed that, although siCTR-treated cells moved persistently towards the cell-free area, siWAS-treated cells migrated in all directions, including parallel to the monolayer free edge (Fig. 6E; Fig. S8G). Cell velocity in the absence of WASp was decreased by ∼60% (Fig. 6D). Furthermore, the migration direction of follower cells (second row of cells from the wound) was also affected (Fig. S8G) indicating a loss of collective coordination of polarity potentially through diminished junctional force transmission between leading and following cells (Carvalho et al., 2019). Based on this, we conclude that human endothelial WASp, like its zebrafish homologue, is essential for directional cell migration.

F-actin and PECAM1 organization were also affected upon depletion of WASp. The characteristic branched actin networks associated with adherens junctions were less prominent in siWASp cells, suggesting a decrease in the formation junction associated intermediated lamellipodia (JAIL), which are essential for cell migration in endothelial monolayers (Cao et al., 2017). By performing WCA and time-lapse imaging in transduced HUVECs with LifeAct-mCherry and VE-Cadherin-GFP reporters, we found that siWAS depletion reduced the frequency of JAILs by 50% (Fig. S8H), suggesting a possible mechanism behind impaired cell migration. Finally, depletion of WASp in HUVECs impaired junctional localization of PECAM1 in monolayers (Fig. 6F). Conversely, knockdown of PECAM1 (siPECAM) in HUVECs impaired oriented cell migration (Fig. S8K,L). Together, these results demonstrate a previously undiscovered role of WASp in the regulation of junctional actin and PECAM1 localization, required for coordinated directional migration of endothelial cells.

## DISCUSSION

**Fig. 7.**
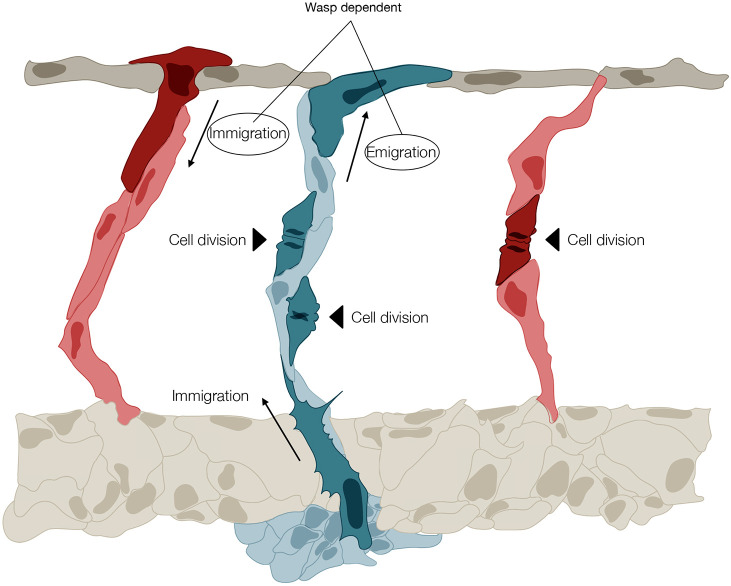
**Illustration of the principal morphodynamic behaviours that differentially control regular vessel diameter formation in arteries and veins of the zebrafish trunk.** A vISV (blue) is shown with two ECs undergoing division (black arrowhead), and with one cell immigrating from the PCV (the PCV vessel is not fully illustrated) and one cell emigrating to the DLAV (all morphodynamic events are highlighted in dark blue). Note that in the vISV, emigration and immigration are balanced, and cell numbers are increased by proliferation. Two arteries (red) are shown either side of the vISV. On the left, the aISV receives a cell by immigration from the DLAV (dark red). On the right, the aISV increases its cell number by cell division (dark red). In aISVs, these two processes are depicted as alternatives, as they seldom coincide. The directional movement of EC from vISV to the DLAV, and from the DLAV to aISV, requires WASp.

The present study provides two major insights into the mechanisms of vascular patterning. First, it predicts that diameter control in the zebrafish trunk is achieved through a balance of directional migration patterns and proliferation rates that differ remarkably between future arteries and veins throughout the remodelling process. Accordingly, the uniform distribution of endothelial cells between the vessels is determined by a balance between the rate of proliferation and the immigration and emigration of ECs to and from the neighbouring segments. Our work demonstrates that these two parameters, cell division and exchange, are not independent, but strongly correlated in a manner that depends on WSS. This applies to both arteries and veins; however, arteries primarily rely on immigration of cells, whereas veins prominently rely on proliferation of EC. In sum, our data demonstrate that the exchange of ECs from proliferative future vISVs into the DLAV, and from the DLAV into less proliferative aISVs appears to be a tightly regulated morphodynamic cell behaviour establishing regular vessel diameter (Fig. 7). Second, our work identifies a molecular mechanism responsible for this differential behaviour depending on junctional F-actin assembly through the NPF WASp. This is surprising as WASp was previously believed to be expressed primarily in immune cells and associated to immune and blood deficiencies (Thrasher and Burns, 2010). WASp is an integral part of the molecular machinery responsible for motility of the immune cells. Previous studies in the cells of patients with the Wiskott-Aldrich syndrome and in WASp-knockout mice revealed perturbed oriented migration (Snapper et al., 2005; Zicha et al., 1998). In agreement with these observations, our results show that WASp function is conserved in ECs. Impaired expression of endothelial Wasb results in disrupted migration patterns, which in turn causes aberrant connections, dramatic increase and irregularity of vein diameter, and a formation and persistence of arteriovenous shunts, which phenotypically and haemodynamically resemble human vascular malformations (Cox et al., 2014; Folkman and D'Amore, 1996). We propose that Wasb-deficient ECs exhibit an impaired migration response that prevents the reorientation required during vessel remodelling. We believe that this phenomenon is analogous to the loss of navigation accuracy in leukocyte and macrophage Wasb-deficient cells (Cvejic et al., 2008). The principle of balanced directional migration provides a new conceptual framework to understand the emergence of vascular malformations and suggests that proliferation alone is unlikely to be the exclusive driver of such pathologies.

Notably, although leukocytes migrate as individual cells, the endothelial cells migrate within a monolayer with continuous cell junctions. Such behaviour requires dynamic junctional remodelling to allow cells to rearrange and migrate collectively. We show that WASp-dependent actin polymerization is necessary for junctional accumulation of PECAM1. Similar to VE-cadherin, PECAM1 has been shown to be involved in force transmission in response to shear stress (Conway and Schwartz, 2015) and we suspect that it might be involved in the balancing of forces that cells exert on one another during the development of the vascular network.

In this study we report the first analysis of WASp in a cellular context other than the immune system, challenging the view of restricted molecular pathways associated to F-actin regulation and migration of haematopoietic cells. Instead, we demonstrate that these mechanisms are at work in cells of haemangioblast origin, providing potential new avenues for the treatment of vascular diseases by focusing on selective targets that control endothelial actin regulation. Given the identified potential for WASp loss-of-function to drive AVM formation, future work will need to establish whether vascular pathologies in Wiskott-Aldrich syndrome patients may extend beyond the rare case reports of aneurysms and vasculitis.

## MATERIALS AND METHODS

### Zebrafish husbandry and transgenic lines

*Danio rerio* (zebrafish) were raised and staged as previously described (Conway and Schwartz, 2015). The following transgenic lines were used: Tg[fli1a:pecam1-EGFP]ncv27 (Conway and Schwartz, 2015) (labels EC junctions), Tg(kdr-l:ras-Cherry)^s916^ (Hogan et al., 2009) (labels EC membrane), Tg(fli1ep:Lifeact-EGFP) (Phng et al., 2013) (labels endothelial F-actin), Tg(fli1:NLS-mCherry) (Heckel et al., 2015) (labels all EC nuclei) and Tg(ve-cad:ve-cadTS) (Lagendijk et al., 2017) (labels cadherin 5). For growing and breeding of transgenic lines, we complied with regulations of the animal ethics committees at MDC Berlin.

### Generation of transgenic *wasb* reporter line

Clone BC053291 from Bioscience was used to amplify *wasb* coding sequence (forward primer, ATGAGTAAGGGTAAGAGTAAGGGACAGGAGAACGTGTCGAGTTCTC; reverse primer, GTCATCCCATTCATCATCTTCTTCTTCTTC). Second PCR was performed to include attB sequences (forward primer, GGGGACAAGTTTGTACAAAAAAGCAGGCTTAATGAGTAAGGGTAAGAGTAAGGGACAG; reverse primer, GGGGACCACTTTGTACAAGAAAGCTGGGTTGTCATCCCATTCATCATCTTCTTCTTCTTCG). This construct was cloned in a pDONRTM221 plasmid. The expression clone was generated with Multisite Gateway cloning (Life Technologies). Zebrafish embryos were injected at the one-cell stage with 100 pg of *Tol2* mRNA and 15 pg of plasmid DNA pTol2-UAS-wasb-mCherry.

### Live imaging zebrafish embryos

Embryos were anaesthetized in 0.007% tricaine (MS-222, Sigma-Aldrich), mounted in a 35 mm Sarstedt (#82.1473) Petri dish using 0.8% low melting point agarose (Sigma-Aldrich) and bathed in Danieau's buffer containing 0.007% tricaine and 0.003% PTU. Time-lapse imaging was performed using an upright 3i spinning-disc confocal using a Zeiss Plan-Apochromat 20×, 40× or 63×/1.0 NA water-dipping objective and heating chamber image. Processing was performed using Fiji software (Schindelin et al., 2012). Frame rate was 10 min.

### Morpholino knockdown

Morpholinos against *wasb* were used as previously described (Cvejic et al., 2008; Jones et al., 2013). *wasb* morpholino sequence: 5′-GCCCTTTGCTTTTGCCTTTGCTCAT (molecular weight: 8334). Control morpholino sequence: 5′-CCTCTTACCTCAGTTACAATTTATA (molecular weight: 8328). *gata1* morpholino sequence: 5′-CTGCAAGTGTAGTATTGAAGATGTC (molecular weight: 8538.15, working amount of *MO-gata1* was 34 ng for all experiments). All morpholinos were produced by Gene Tools.

### Imaging of HUVECs

Time-lapse imaging was performed using a Carl Zeiss LSM780 inverted microscope with a Plan-Apochromat 20×/0.8 at 37°C under 5% CO_2_. Imaging of fixed samples was performed using a Carl Zeiss LSM700 upright microscope with a Plan-Apochromat 20×/0.8. Live imaging was recorded with a frame rate of 10 min.

### Scratch wound assay

Cells were re-plated into a scratch wound assay device (IBIDI) 24 h after siRNA transfection. On the following day, a cell free gap of 500 µm was created by removing the insert of the device. Live imaging was performed immediately after removing the insert. For cell coverage measurements images were taken immediately after removing the insert and after 16 h using a Leica DMIL LED microscope equipped with a 10×/0.22 NA Ph1 objective and a CCD camera (DFC3000 G). The cell-free area was measured in Fiji and used to calculate the percentage of wound closure at 16 h.

### Immunofluorescence staining

For immunofluorescence in HUVECs, cells were grown in #1.5 coverslips coated with poly-lysine and gelatin 0.2%. At the end of the experiment cells were fixed in 4% paraformaldehyde (PFA) for 10 min, permeabilized in 0.3% Triton X-100 in blocking buffer [3% bovine serum albumin (BSA), 0.05% Triton X-100] for 5 min and blocked in 1% BSA with 20 mM glycine in PBS for 30 min. Primary and secondary antibodies were incubated for 2 h and 1 h, respectively, in blocking buffer. Nuclei labelling was performed by incubating cells with DAPI for 5 min (Life Technologies, D1306) and Alexa Fluor 568 Phalloidin (1/600, Thermo Fisher Scientific, A12380). Primary antibodies used were: human VE-cadherin (1/100, R&D Systems, AF938), human PECAM1 (1/200, Abcam, ab76533), human Ki67 (1:100 Abcam, ab15580).

### Segmentation and tracking of endothelial nuclei

Segmentation and tracking of nuclei from live imaging in zebrafish and cultured HUVECs was performed with Imaris Image Tracking Package (Bitplane). Tracking data was formatted according to community standards for open cell migration data (Gonzalez-Beltran et al., 2020). For each zebrafish the cell tracking data was oriented in three-dimensional space such that the aorta was parallel to the *x*-axis and the DLAV in the positive-*y* half plane. In this way, the *y*-component of the transformed trajectories served as a read-out for the ventral to dorsal positions of the nuclei. In order to determine the orientation of the aorta we used positional data of all nuclei in the aorta over the whole time period and performed linear regression. The tracking was subsequently rotated and translated in three-dimensional space (see https://github.com/wgiese/zebrafish_ec_migration/wiki for details). We extracted ventral to dorsal velocities over the developmental process by calculating the signed displacement in *y*-direction over time windows of 2 h. This process was repeated over the whole time period from 26 hpf to 44 hpf in 10 min time steps for all cell tracks. In HUVECs, cell trajectory data was aligned such that the *y*-axis followed the outer edge of the scratch-wound, whereas the cell-free space is on the positive-*x* half plane. We calculated the effective speed as the quotient of the distance from start to end point and the tracking time. Furthermore, we determined the directionality of each trajectory from the vector pointing from start to end point and calculated the angle with the *y*-axis. The data that support the findings of this study are available at https://doi.org/10.5061/dryad.2fqz612q6 (Rosa et al., 2022).

### Crispr-Cas9 knockout

Guide RNA design was performed using ZiFit Targeter software package. RNA guide with sequence TAGGAATGGAGTCTCCAGCATAC was used to target exon 2 of zebrafish *wasb*. Cas9 mRNA and guide RNA was injected in one-cell-stage embryos of Tg(fli1ep:Lifeact-EGFP);Tg(fli1:NLS-mCherry).

### Cell dissociation for FACS

Dechorionation of zebrafish embryos was performed by gently pipetting embryos up and down in a solution of Danieau's buffer containing pronase. After dechorionation, embryos were transferred to a calcium-free Ringer’s solution [116 mM NaCl, 2.9 mM KCl, 5 mM HEPES (pH 7.2)]. Yolk was removed by gently pipetting. Supernatant was removed by centrifugation (720 rcf, Eppendorf 5804 R) for 5 min at 4°C. Dissociation was performed by re-suspension in liberase solution (0.8 mg/ml liberase in DPBS) at 28.5°C for 10 min. To stop dissociation, samples were placed on ice and added to a solution with 1-2% CaCl_2_ and 5-10% fetal bovine serum (FBS). Finally, we centrifuged for 5 min (720 rcf) at 4°C, discarded supernatant and resuspended in DPBS with 2 mM of EDTA. FACS was performed in a BD Aria sorter at the MDC flow cytometry facility. Sorted cells were kept at −80°C in Trizol.

### RNA extraction and real-time quantitative RT-PCR

RNA extraction was performed making use of Direct-zolTM RNA MicroPrep (Zymo Research, R2060, R2061, R2062, R2063). First strand cDNA synthesis was performed with Thermo Fisher Scientific RevertAid Reverse Transcriptase kit (Thermo Fisher Scientific, EP0441) with random hexamer primers for cells extracted from zebrafish embryos, and RNeasy plus Mini Kit (Qiagen, 74034) for HUVEC samples. SYBR Green real-time quantitative PCR was performed following SG qPCR Master mix protocol and reagents from Roboklon (EURx Roboklon, E0402-01) for zebrafish samples. Primers used for quantitative RT PCR were as previously published (Cvejic et al., 2008). For HUVEC samples, real-time quantitative PCR was performed using TaqMan reagents (Applied Biosystems).

### *In silico* idealized ISV network

We constructed an idealized model of flow within the ISV network for each zebrafish experiment using summary statistics in order to estimate flow and WSS experienced by ECs. Each vessel network was represented as a graph (i.e. a collection of nodes and edges). Each network consisted of 30 ISVs with an alternating pattern of aISV-vISV. The ISVs were positioned within the network according to mean ISV spacing and ISV length measured from the experiment. Each edge was also assigned a diameter function over time based on mean diameter measurements of each vessel type (DA, DLAV, aISV or vISV). The distinction of aISV or vISV came from end-point analysis, i.e. designating the vessel by which vessel the ISV was connected to at the end of the experiment (either artery or vein).

Boundary nodes were defined at the inlet and outlet of the DA, as well as at each of the terminal ends of the vISVs. Owing to difficultly in imaging and distinguishing the pectoral cardinal vein from the DA, the vein was not included in the network and instead was treated as a flow sink (*P*_PCV_=0 Pa). Pressure at the inlet was estimated from measurements from the DA of adult zebrafish (Hu et al., 2001) (*P*_DA,in_=201.3 Pa). A pressure gradient was prescribed along the aorta in order to maintain forward flow through the vessel over time. We estimated that this gradient dissipated the inlet pressure over a distance over the length of whole fish, *L*_fish_=2 mm, and applied this gradient to the fraction of the aorta included in our flow model,
(1)

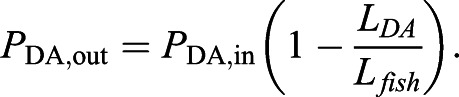
The flow conductance (i.e. the inverse of flow resistance) of each edge was calculated as
(2)

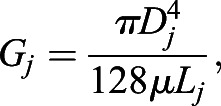
with *D*_*j*_ and *L*_*j*_ as the diameter and length of edge *j* at time point *t*, and μ the dynamic viscosity of blood (μ=0.0035 kg m^−1^ s^−1^). We used a flow balance equation at each node to assemble the linear system of equations for the network, which we can use to solve for the unknown nodal pressures and therefore flow based on vessel conductivity,
(3)

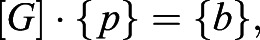
where {*p*} is the array of unknown nodal pressures, [*G*] is network conductivity matrix assembled with edge connectivity and conductivity, and {*b*} is the solution array which enforces the flow balance and contains information on the pressure boundary conditions [see Pries et al. (1990) for a detailed description]. We solved this system of equations for each time point, each time updating [*G*] based on the measurements of vessel diameter obtained from the experiments. Edges with no vessel diameter measurement (i.e. *D_j_*=0) had their conductance set to an infinitesimally small value (∼10^−30^) to keep [*G*] invertible.

We used MATLAB's ‘mldivide’ operator (release 2018b) to solve the linear system of equations for the nodal pressures, with which we could calculate the pressure difference across each vessel segment and thus the flow, *Q*, and wall shear stress, τ, for each edge:
(4)

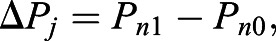

(5)

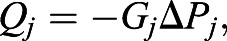

(6)

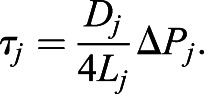


The MATLAB code for flow inference is available at https://github.com/ltedgar-ed/flow_idealised_ISV_network.

### HUVEC gene silencing

For knockdown experiments, HUVECs (Promocell, pool donor) were transfected with SMARTpool: siGENOME siRNAs purchased from Dharmacon (was M-028294-02-0005, non-targeting siRNA Pool 1 #D001206-13-05). Briefly, subconfluent (70-80%) HUVECs were transfected with 25 nM siRNA using Dharmafect 1 transfection reagent following the protocol from the manufacturer; transfection medium was removed after 24 h and experiments were routinely performed on the second day after transfection.

### Computational analysis pipeline

We used the python-based workflow manager Kedro for computational analysis: the code can be accessed on github via https://github.com/wgiese/zebrafish_ec_migration. For all zebrafish and HUVECs experiments we created two key files, which contain metadata on the experimental setup as well as references to cell tracking data. Based on the key files our generic data pipeline allows the linking of datasets comprising cell trajectories, vessel diameters, vessel cell number, cell mitosis and inter-vessel cell migration.

### Statistical analysis

For statistical analysis of cell number, cell divisions and inter-vessel migration distributions we used a modified version of the Kolmogorov–Smirnov test for discrete distributions by using the r-package dgof (Arnold and Emerson, 2011). The analysis of the HUVEC orientation data was performed based on the r-packages CircStats and circular, which provide statistical analysis of periodic data, including mean, standard deviation and Watson's U2 test for circular distributions.

## Supplementary Material

Supplementary information
