# Supplementary material for: WASp controls oriented migration of endothelial cells to achieve functional vascular patterning
Source: Development. 2022 Feb 1;149(3):dev200195. doi: 10.1242/dev.200195 (PMC8918813; doi:10.1242/dev.200195)
Supplement: Supplementary information [file develop-149-200195-s1.pdf]

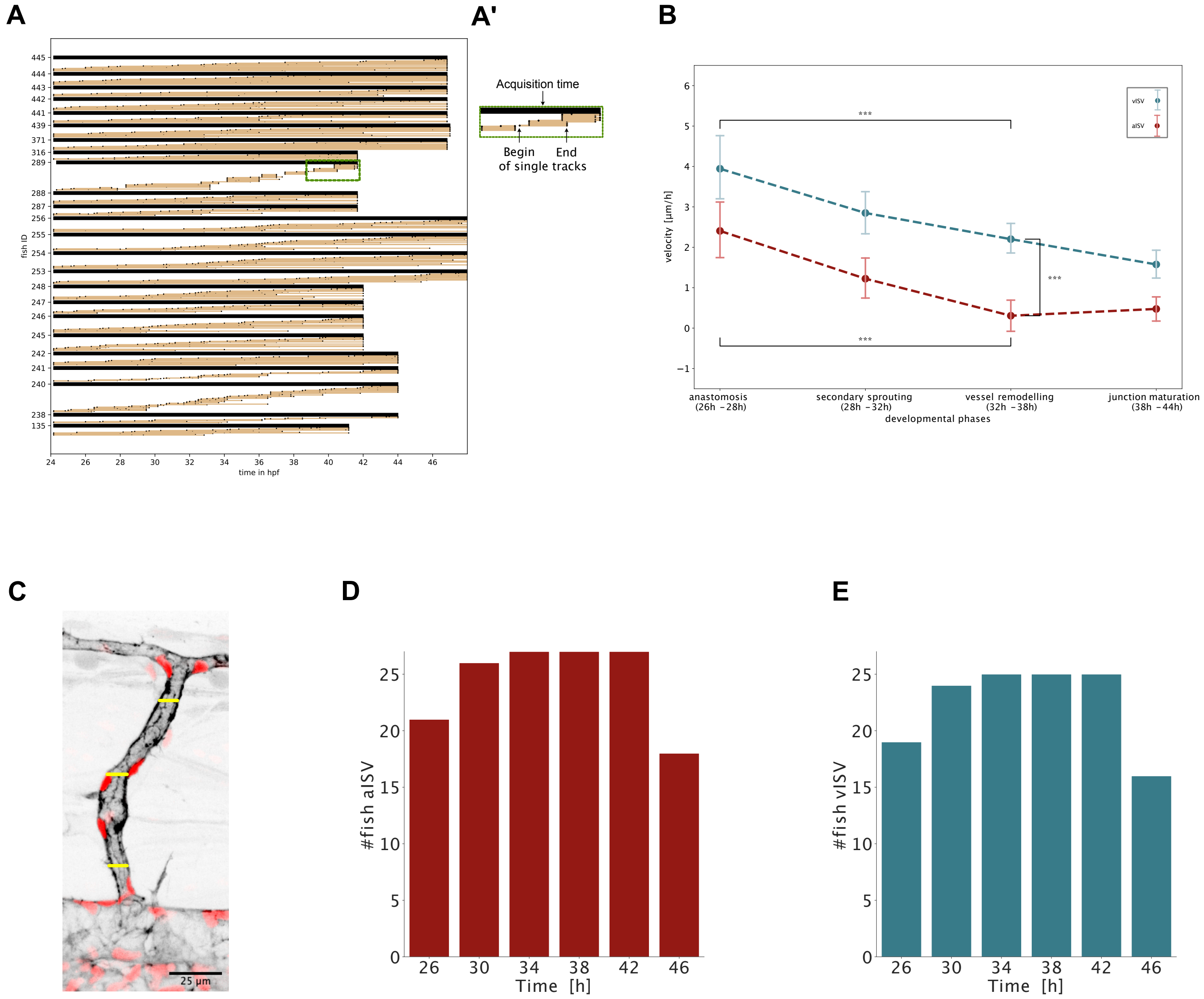

Fig. S1. Sample size and statistical tests for MO-Control. A, Abundance of nuclei tracks in MO-Ctr. Each number on the y-axis represents a unique experiment ID, black bars indicate start end time of image acquisition for each experiment, while **A',** each yellow line represents unique nuclei track with start and end time. **B**, Average velocity for different developmental phases in MO-Ctr, velocities were calculated in 2h intervals and pooled according to the time intervals given below the developmental phases. For each developmental phase 95% confidence intervals of the mean velocities are shown, Welch's t-test was used to test for statistical significance during vessel remodelling between aISV and vISV cell velocities, and, additionally, between anastomosis and remodelling for vISV and aISV cell velocities separately. **C**, Illustration for the measurement of ISV diameter in different ISV regions of MO-Ctr. **D** and **E**, Fish sample count for each time point with diameter measurements in MO-Ctr

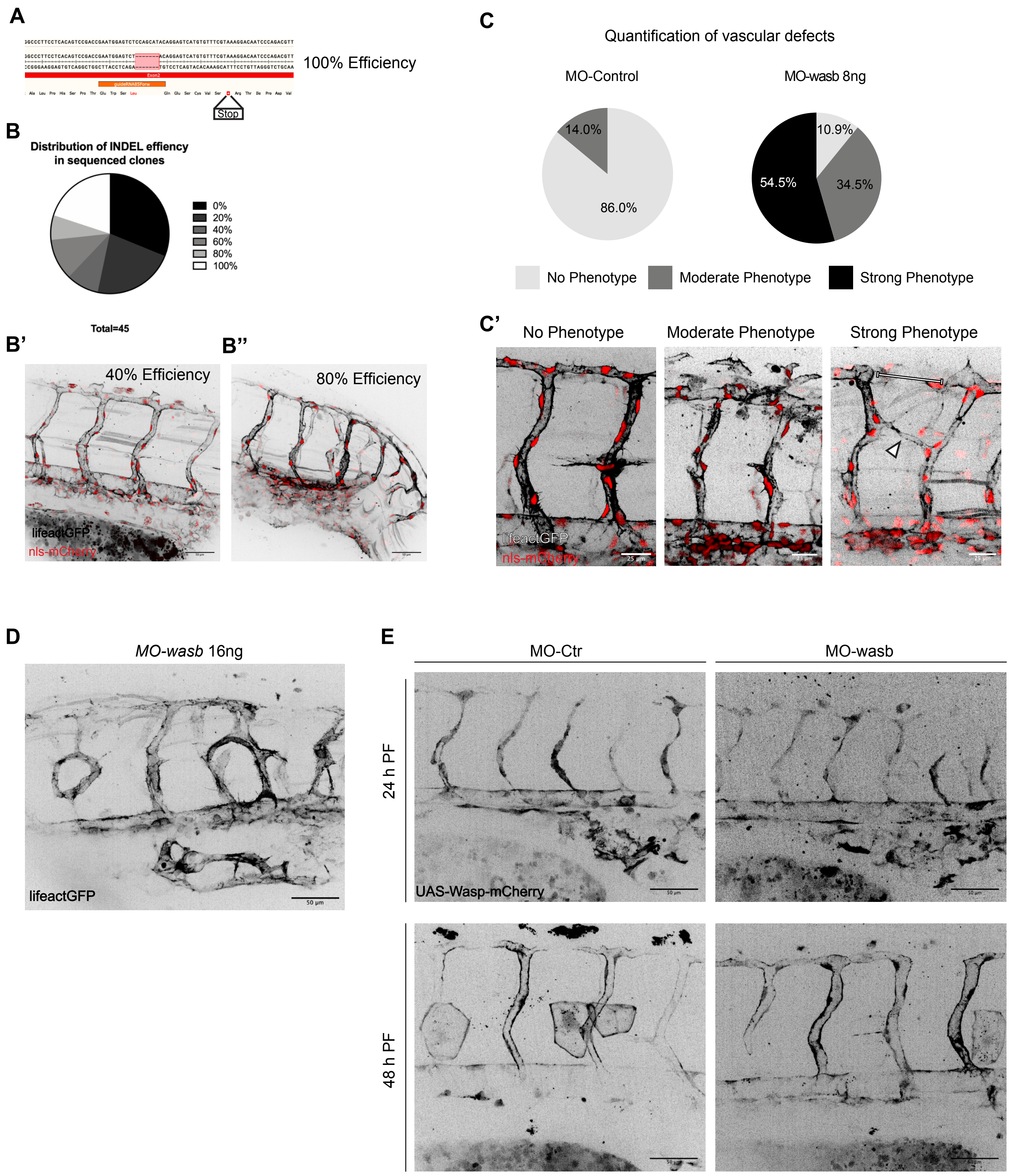

Fig. S2. Vascular morphology is dependent of WASP. A, Deletion in Exon 2 of *wasb* gene in A F0 Crispr-Cas9 *D. rerio* embryo with 100% distribution of InDels. **B**, TOPO Cloning of exon 2 from *wasb* gene was transformed in competent *E. coli*. 5 random single colonies were selected for sequencing. Distribution of InDels was measured on the ratio of sequences from 5 colonies. 0 in 5- 0%; 1 in 5- 20%; 2 in 5- 40%; 3 in 5- 60%; 4 in 5- 80%; 5 in 5- 100%. **C** Quantification of vascular phenotype in *MO-Ctr* and *MO-wasb* injected with 8 ng of morpholino. **C'**, Examples of trunk vasculature in embryos with no, moderate and strong phenotype. **D**, Vascular morphology of (fliep:lifeactGFP) embryos injected with 16 ng of wasb morpholino. **E**, *MO-ctr* and *MO-wasb* (fliep-GAL4:wasb-mCherry) embryos. Total number of embryos used (*MO-Ctr* N=10, *MO-wasb* N=14).

Development • Supplementary information

Supplemental Figure 3

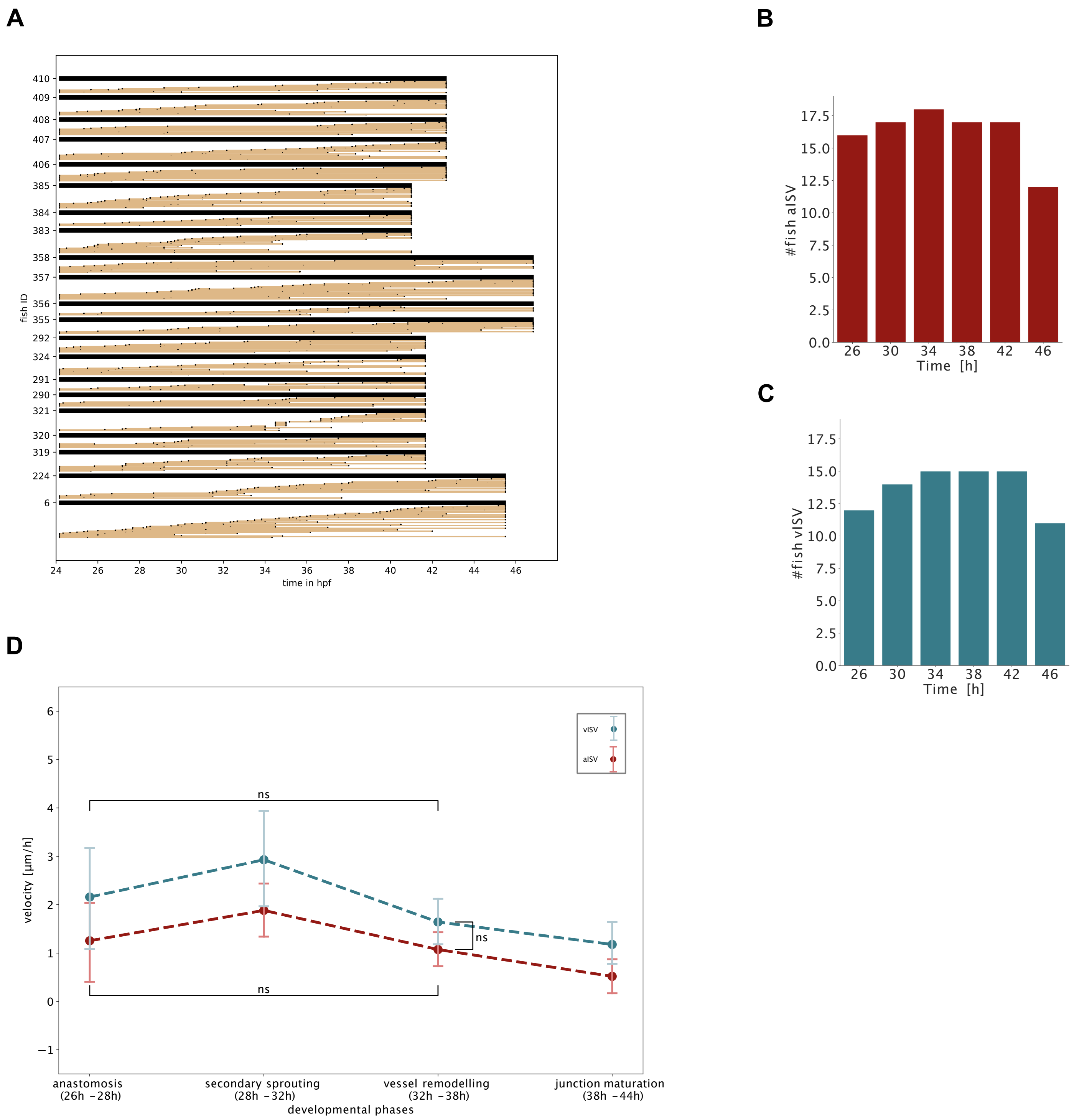

Fig. S3. Sample size and statistical tests for MO-wasb. A, Abundance of nuclei tracks in MO-wasb. Each number on the y-axis represents a unique experiment ID, black bars indicate start end time of image acquisition for each experiment, while each yellow line represents unique nuclei track with start and end time. **B and C,** Fish sample count for each time point with diameter measurements in MO-wasb. **D,** Average velocity for different developmental phases in MO-wasb, velocities were calculated in 2h intervals and pooled according to the time intervals given below the developmental phases. For each developmental phase 95% confidence intervals of the mean velocities are shown, Welch's t-test was used to test for statistical significance during vessel remodelling between aISV and vISV cell velocities, and, additionally, between anastomosis and remodelling for vISV and aISV cell velocities separately.

Development • Supplementary information

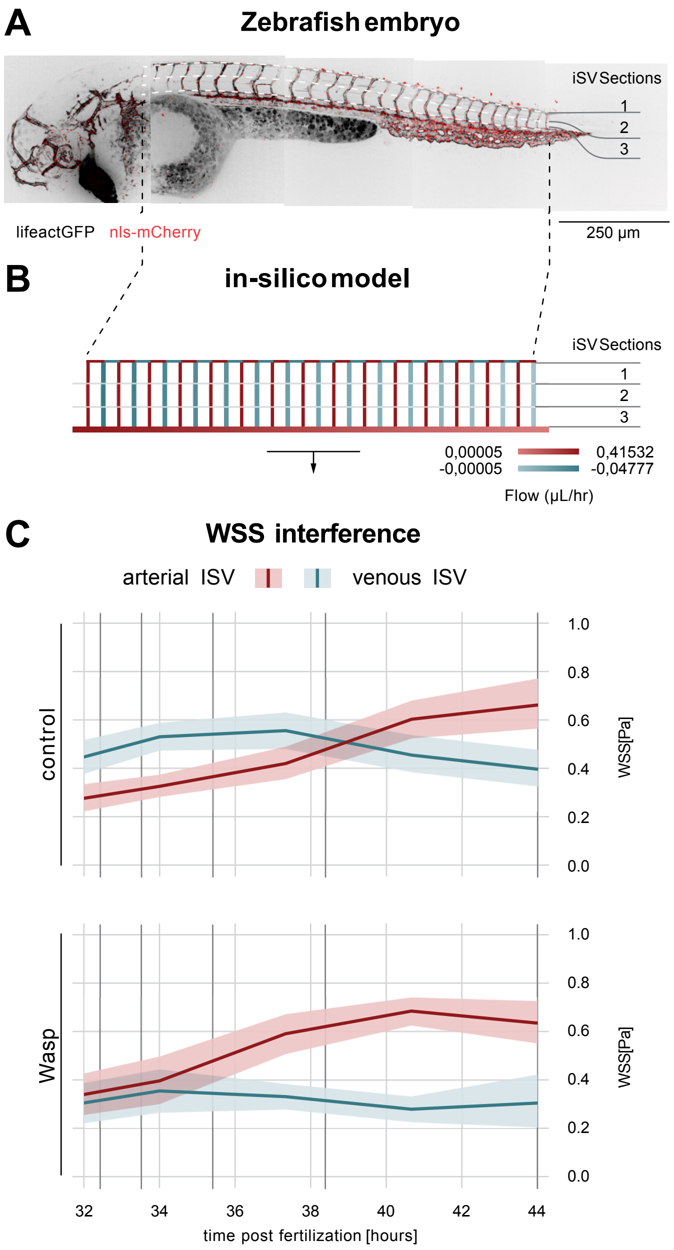

Fig. S4. WSS progression in ISVs is affected in MO-wasb embryos. A, Whole Tg[(fli1ep:Lifeact-EGFP); Tg(fli1:NLS-mCherry)] 48 hour PF embryo with F-actin label in black and EC nuclei in red. ISV Sections 1-3 mark the region of diameter measurements across development. **B**, *In silico* model made of 30 ISV (further details see methods section). Black bar with arrow highlights the region of 3 aISV and 3 vISV used to follow WSS inference across time depicted in **C**. **C,** WSS inference of aISV and vISV in *MO-Ctr* and *MO-wasb* conditions.

Development • Supplementary information

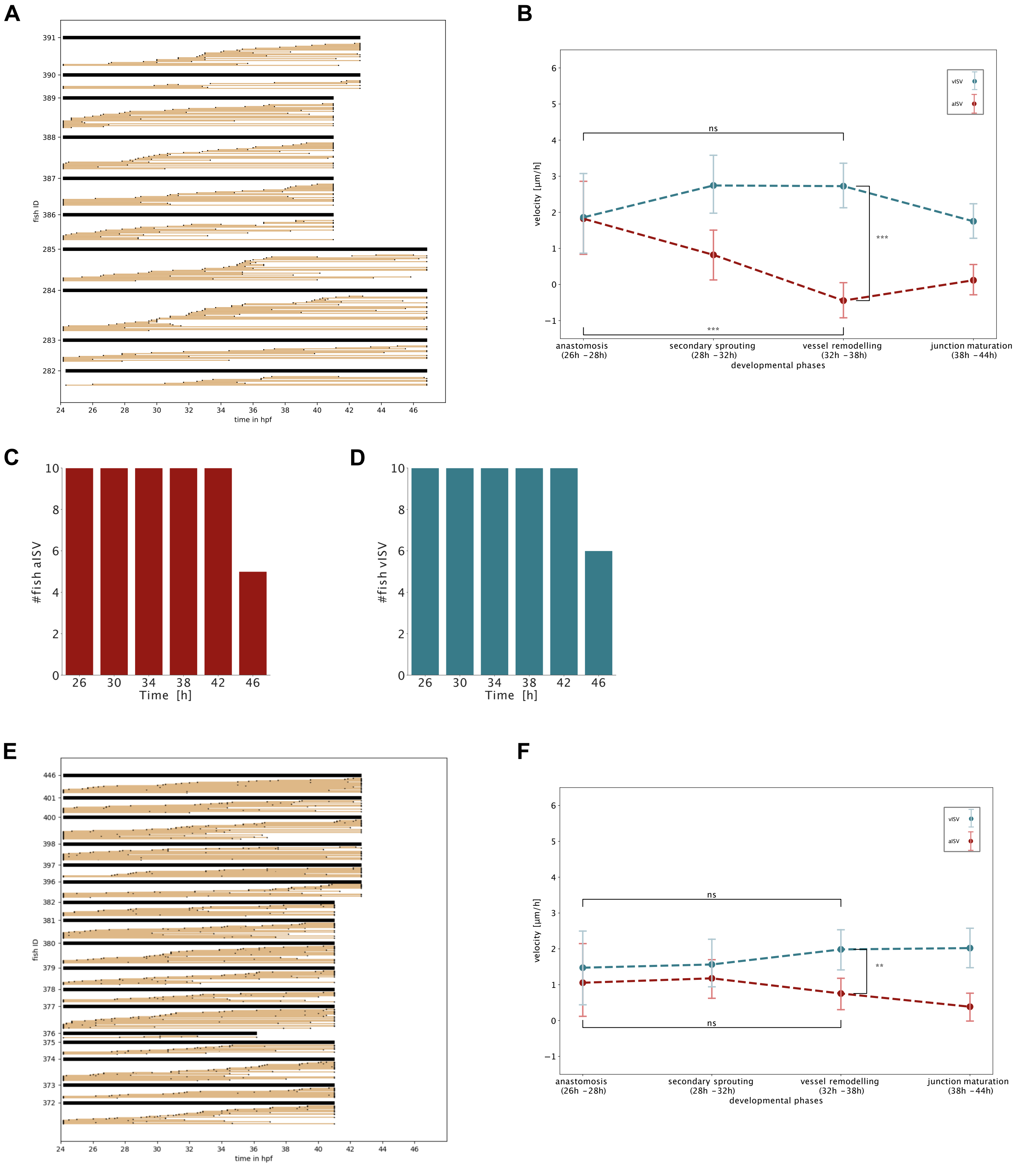

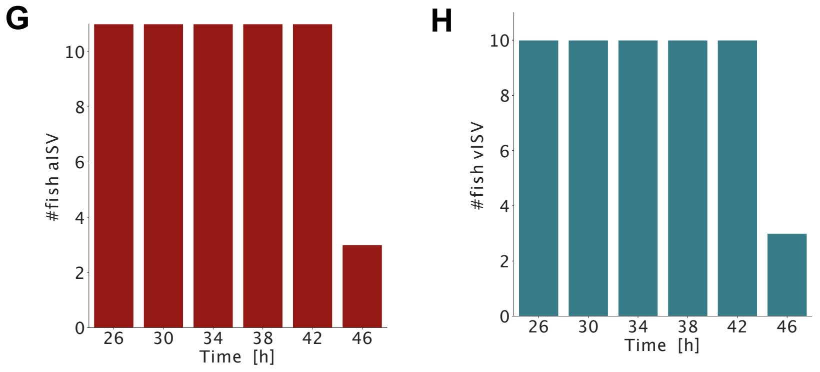

I

Statistical comparison MO-gata1 vs MO-gata1/wasb

Analysis Group 1	Analysis Group 2	Vessel type	Dev. Phase	t-statistic	p-value	Stat. Test
MO-gata1	MO-gata1/wasb	aISV	anastomosis (26h - 28h)	1.0483	0.2964	Welch's t-test
MO-gata1	MO-gata1/wasb	aISV	secondary sprouting (28h - 32h)	-0.7728	0.4402	Welch's t-test
MO-gata1	MO-gata1/wasb	aISV	vessel remodelling (32h - 38h)	-3.4594	0.0006	Welch's t-test
MO-gata1	MO-gata1/wasb	aISV	junction maturation (38h - 44h)	-0.8856	0.3763	Welch's t-test
MO-gata1	MO-gata1/wasb	vISV	anastomosis (26h - 28h)	0.5075	0.6133	Welch's t-test
MO-gata1	MO-gata1/wasb	vISV	secondary sprouting (28h - 32h)	2.1581	0.0321	Welch's t-test
MO-gata1	MO-gata1/wasb	vISV	vessel remodelling (32h - 38h)	1.8276	0.0684	Welch's t-test
MO-gata1	MO-gata1/wasb	vISV	junction maturation (38h - 44h)	-0.7390	0.4604	Welch's t-test

Fig. S5. Sample size and statistical tests for MO-gata1 and MO-gata1/wasb. A, Abundance of nuclei tracks for MO-gata1. Each number on the y-axis represents a unique experiment ID, black bars indicate start end time of image acquisition for each experiment, while each yellow line represents unique nuclei track with start and end time. **B**, Average velocity for MO-gata1 during different developmental phases, velocities were calculated in 2h intervals and pooled according to the time intervals given below the developmental phases. For each developmental phase 95% confidence intervals of the mean velocities are shown, Welch's t-test was used to test for statistical significance during vessel remodelling between aISV and vISV cell velocities, and, additionally, between anastomosis and remodelling for vISV and aISV cell velocities separately. **C** and **D**, Fish sample count for each time point with diameter measurements. **E** Abundance of nuclei tracks for MO-gata1/wasb. F, Average velocity for MO-gata1/wasb during different developmental phases. **G** and **H** Fish sample count for each time point with diameter measurements. **I**, Statistical comparison between *MO-gata1* and *MO-gata1/wasb* at different developmental times in regards to aISV and vISV.

Development • Supplementary information

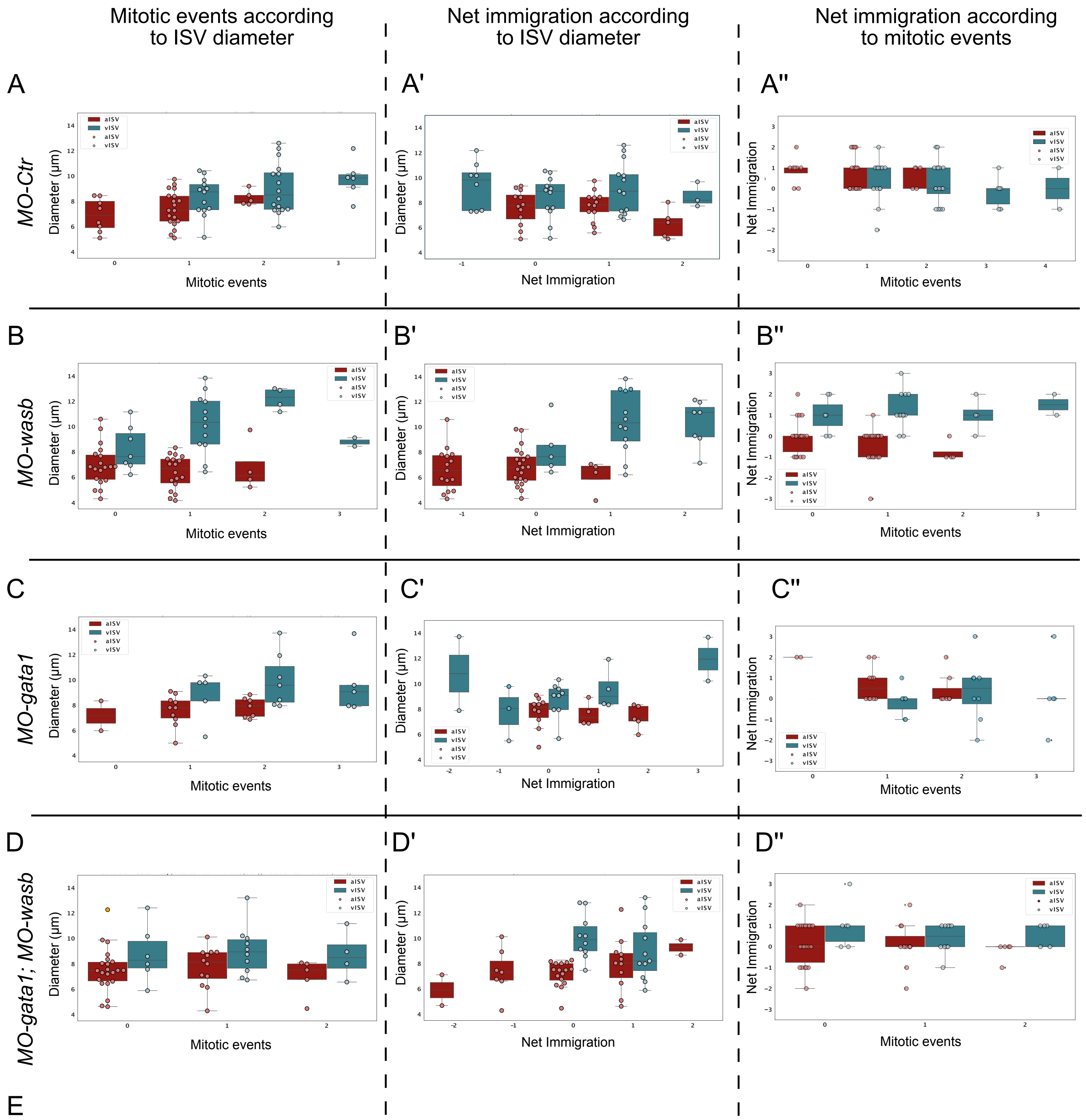

E

Spearman Correlation						
	Diameter vs Mitotic events		Diameter vs Net Immigration		Net Immigration vs Mitotic events	
$aISV_{MO\text{-}Ctr}$	0.312	+	-0.240	-	-0.126	-
$vISV_{MO\text{-}Ctr}$	0.328	+	0.021	n.a	-0.200	-
$aISV_{MO\text{-}wasb}$	-0.039	n.a	-0.059	n.a	-0.259	-
$vISV_{MO\text{-}wasb}$	0.413	+	0.292	+	-0.100	-
$aISV_{MO\text{-}gata1}$	0.141	+	-0.254	-	-0.177	-
$vISV_{MO\text{-}gata1}$	0.043	n.a.	-0.305	-	-0.098	n.a
$aISV_{MO\text{-}gata1;wasb}$	-0.006	n.a.	0.341	+	-0.149	-
$vISV_{MO\text{-}gata1;wasb}$	0.153	+	-0.129	-	-0.126	-

Development • Supplementary information

Fig. S6. Cross-correlation analysis of *MO-Ctr, MO-wasb, MO-gata1* and *MO-gata1/wasbl.* **A-A”** *MO-Ctr*, **A**, Final vessel diameter comparison with mitotic events. **A’**, Final vessel diameter comparison with Net immigration (Net Immigration = $EC_{immigration} – EC_{emigration}$). **A”**, Net Immigration comparison with Mitotic events. **B-B”** *MO-wasb*. **B**, Final vessel diameter comparison with mitotic events. **B’**, Final vessel diameter comparison with Net immigration (Net Immigration = $EC_{immigration} – EC_{emigration}$). **B”**, Net Immigration comparison with Mitotic events. **C-C”** *MO-gata1*. **C**, Final vessel diameter comparison with mitotic events. **C’**, Final vessel diameter comparison with Net immigration (Net Immigration = $EC_{immigration} – EC_{emigration}$). **C”**, Net Immigration comparison with Mitotic events. **D-D”** *MO-gata1/wasb*. **D**, Final vessel diameter comparison with mitotic events. **D’**, Final vessel diameter comparison with Net immigration (Net Immigration = $EC_{immigration} – EC_{emigration}$). **D”**, Net Immigration comparison with Mitotic events. **E**, Spearman Correlation of comparisons. **+** positive correlation**, -** negative correlation, **n.a.** no association.

Development • Supplementary information

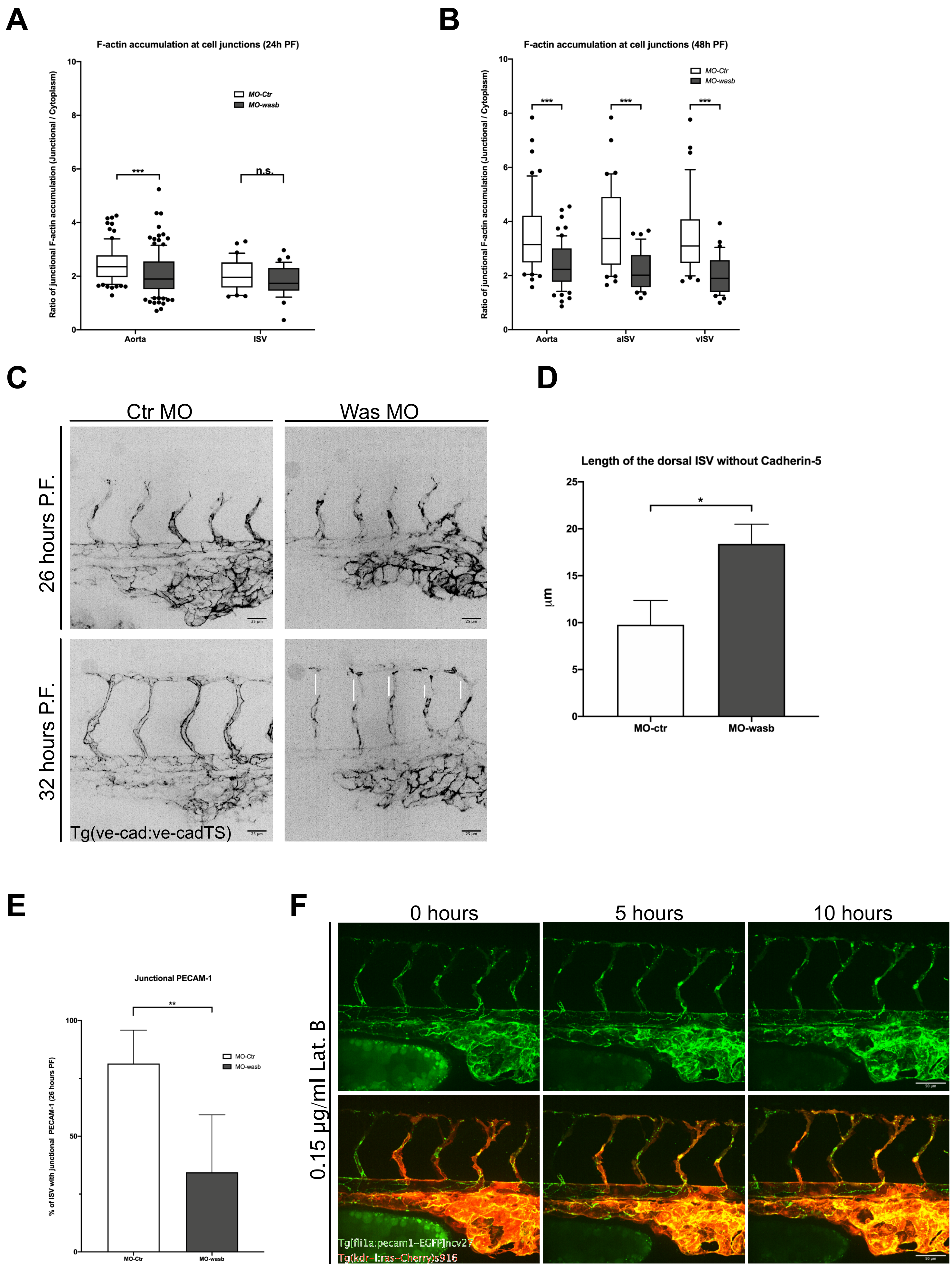

Development • Supplementary information

Fig. S7. Wasb regulates actin organization and PECAM localization. A, Average ratio intensity of Lifeact-GFP (junctions/cytoplasm) 24h PF MO-ctr and MO-wasb. A line was drawn along junctions and along the cell cytoplasm (of the same length and width). The average intensity of each line was normalized against background signal. **B**, Average ratio intensity of Lifeact-GFP (junctions/cytoplasm) 48h PF MO-ctr and MO-wasb. **C**, Cadherin-5 expression and localization in MO-Ctr and MO-wasb embryos. **D**, Quantification of dorsal ISV without Cadherin-5. **E**, Percentage of ISV with junctional PECAM in MO-Ctr and MO-wasb embryos. **F**, 0,15 µg/ml Latrunculin B treated *Tg(fli1a:pecam1-EGFP*^{ncv27}*;* kdr-l:ras-Cherry)^{s91} embryos.

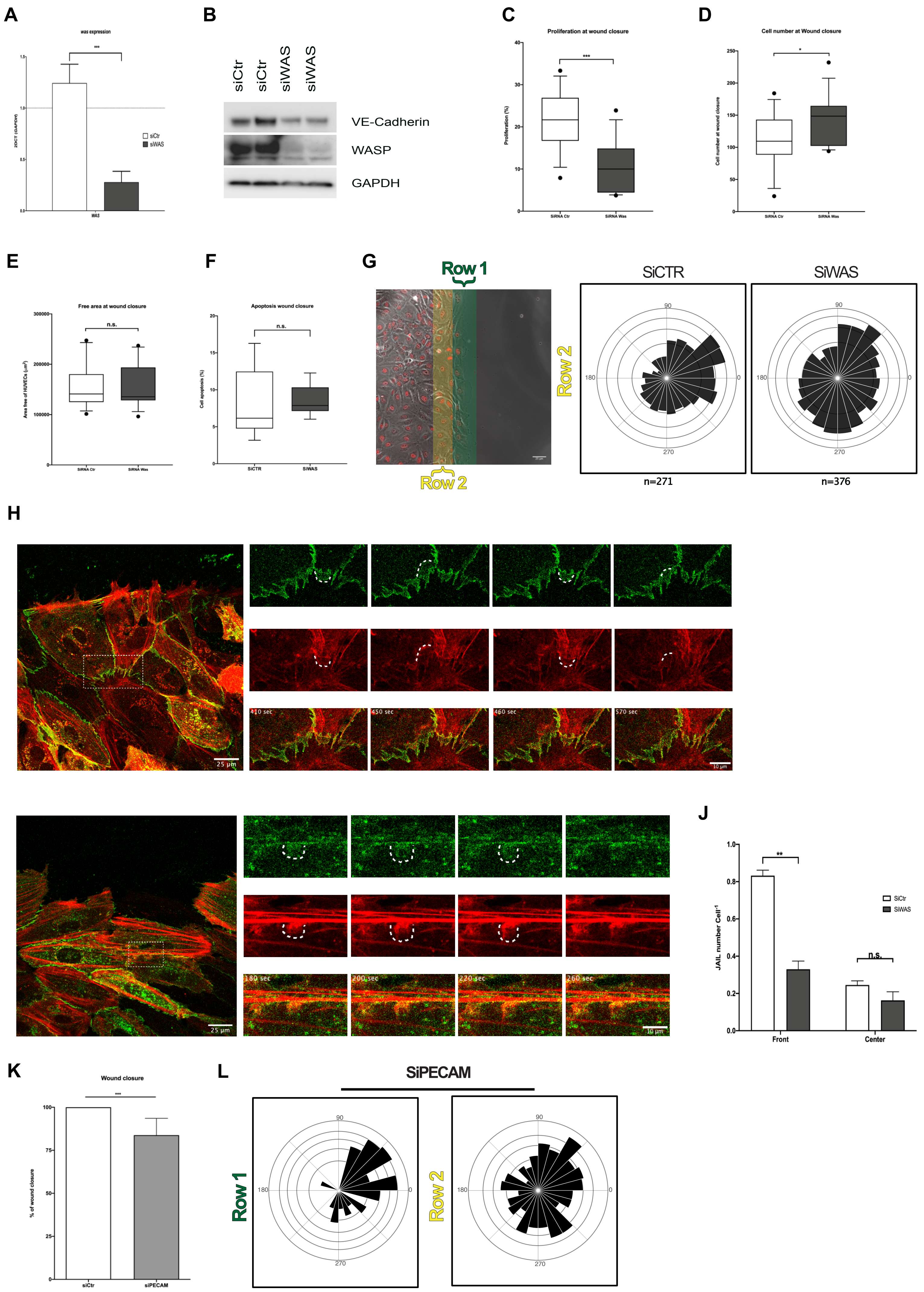

Development • Supplementary information

Fig. S8. WASp is required for oriented cell migration in human ECs. A, HUVECs was expression in SiCtr and SiWAS treated cells. **B**, Western blot against VE-Cadherin, WASp and GAPDH from protein extracts of SiCTR and SiWAS. **C**, Quantification of cell proliferation at wound closure for SiCTR and SiWAS. **D**, Quantification of cell number at the wound closure for SiCTR and SiWAS. **E**, Quantification of free area at the wound closure for SiCTR and SiWAS. **F**, Quantification of apoptotic events at the wound closure for SiCTR and SiWAS. **G**, Nuclei tracking of HUVECs treated with SiCTR and SiWASP at the very edge of wound (ROW1) the line of cells just before (ROW2). Rosette graph showing the prevalent cell direction during wound closure in SiCTR and SiWAS. Comparison between graphs pvalue<0,001 (Watson Two-Sample test for homogeneity). **H**, WCA live imaging of SiCTR and SiWAS treated cells transduced with Lifeact-mCherry and VE-Cadherin- GFP and quantification of Jail events per cell. **K**, quantification of wound closure at 16 hrs for SiPECAM treated HUVECs. **L**, Rosette graph showing the prevalent cell direction during wound closure in SiPECAM.

Development • Supplementary information

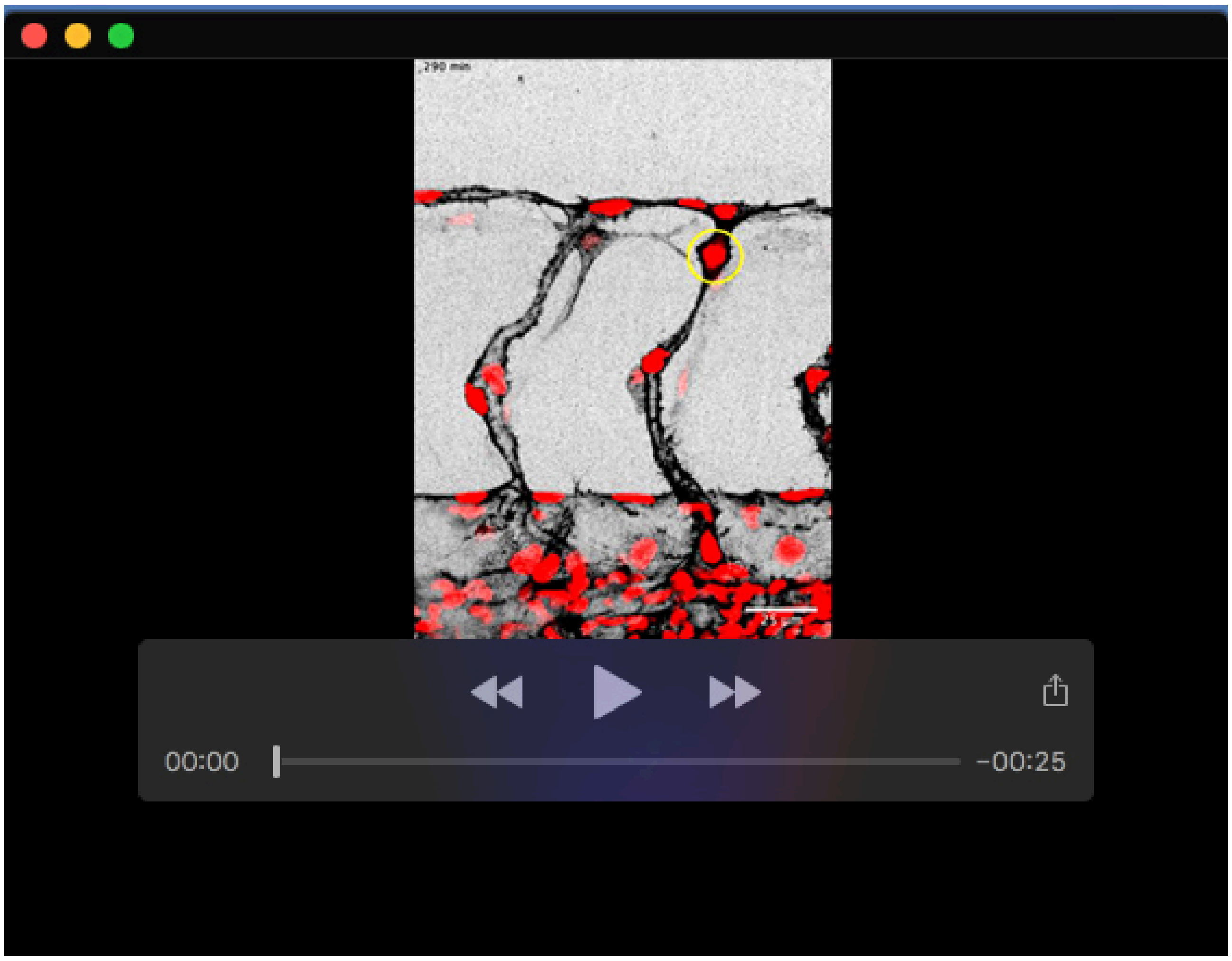

Movie 1. Time-lapse of 26 hours PF trunk vasculature of *MO-control* Tg[(fli1ep:Lifeact-EGFP); Tg(fli1:NLS-mCherry)] embryo. Time frame 10 min. White arrowhead (min 30) - anastomosis. Black arrow head (min 140) - secondary sprouting. Yellow circle (min 280, 370) - mitosis (strong cortical F-actin accumulation). Blue arrowhead (min 310) - emigration to the DLAV. Red arrowhead (min 440) - immigration from DLAV.

Development • Supplementary information

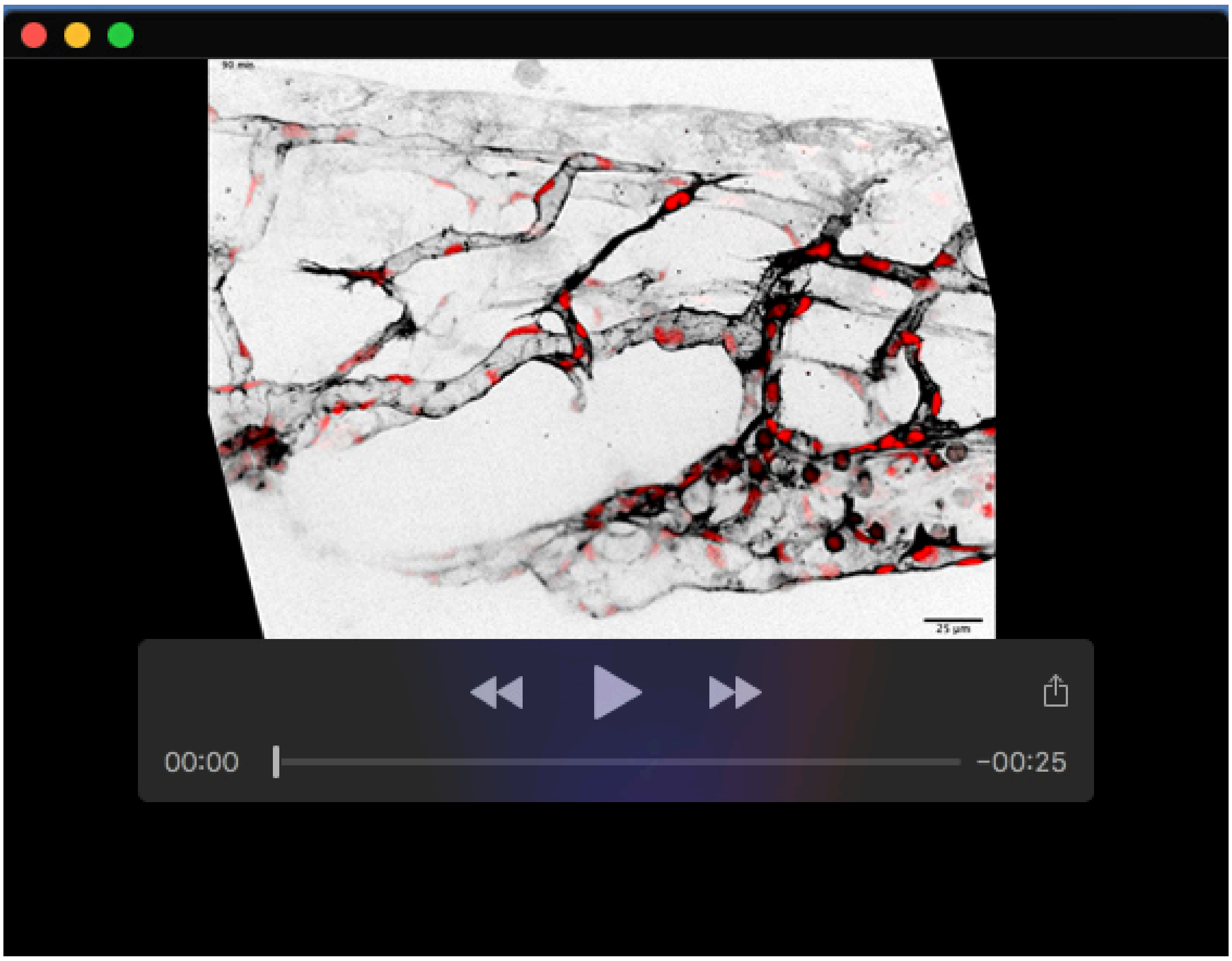

Movie 2. Time-lapse of 44 hours PF trunk vasculature of F0 *was* crispant (100% deletion) Tg[(fli1ep:Lifeact-EGFP); Tg(fli1:NLS-mCherry)] embryo. Time frame 10 min.

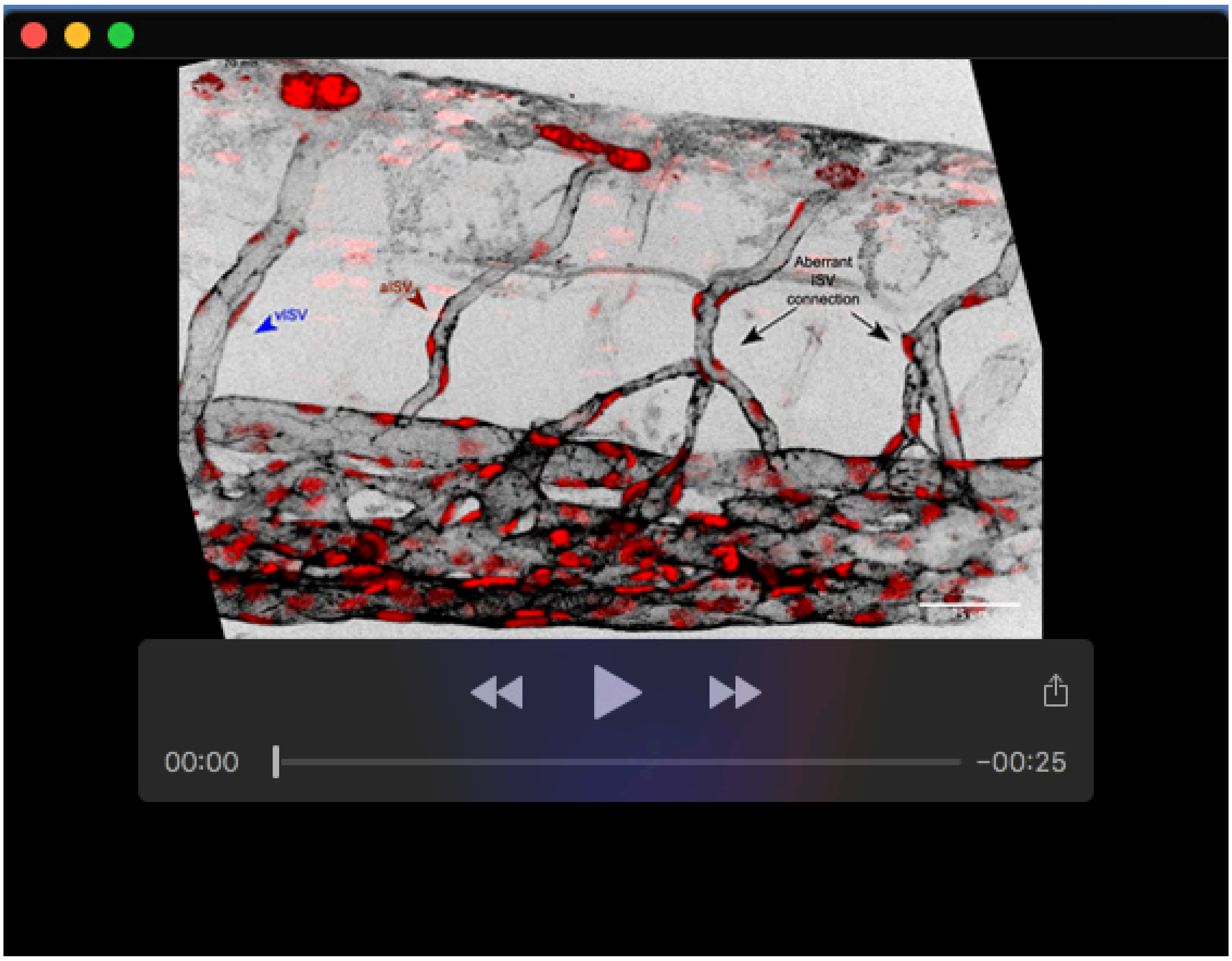

Movie 3. Time-lapse of 5 days PF trunk vasculature of *MO-wasb* Tg[(fli1ep:Lifeact-EGFP); Tg(fli1:NLS-mCherry)] embryo. Blue arrowhead- vISV. Red arrowhead aISV. Black arrows- vessel malformations. Time frame 10 min.

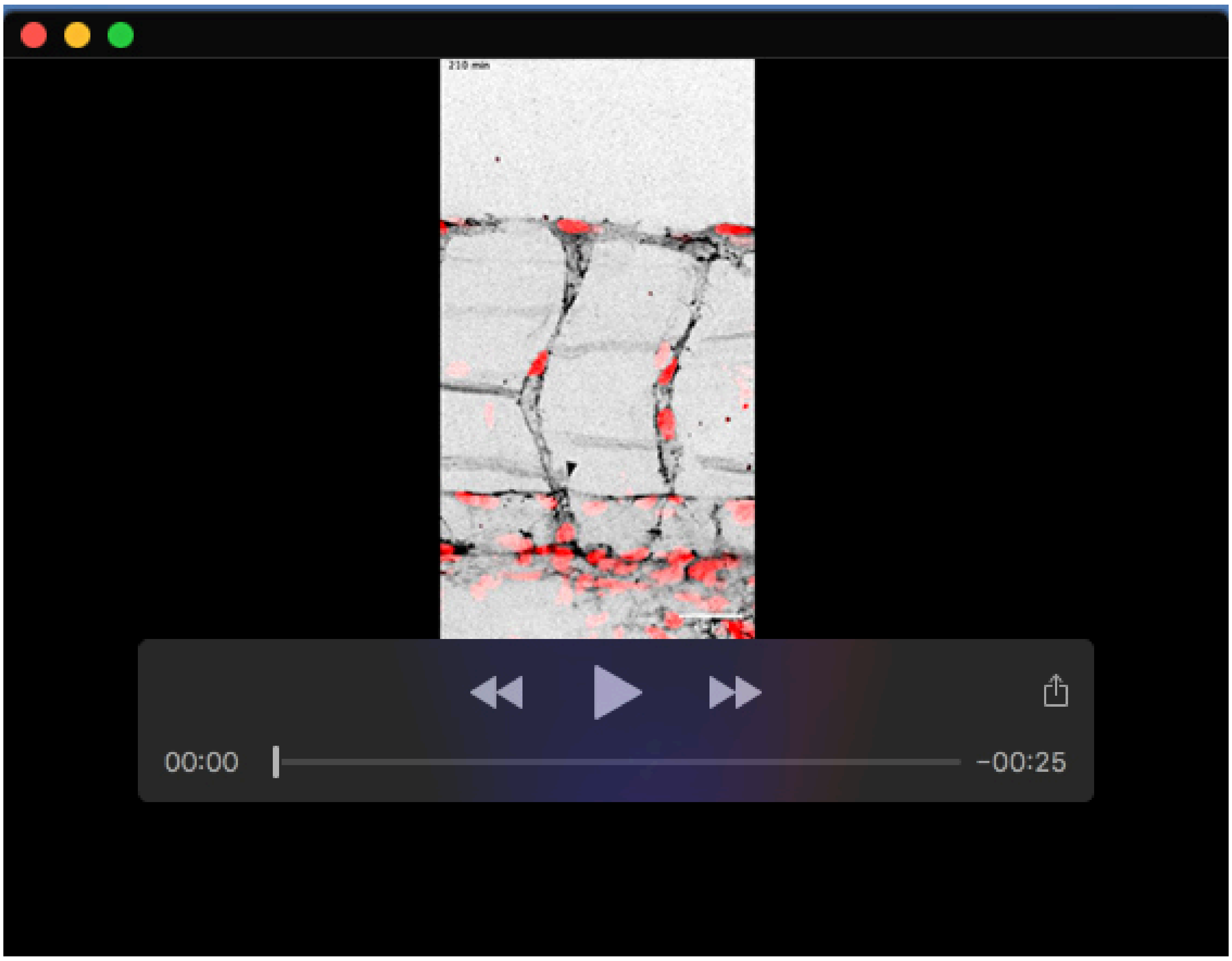

Movie 4. Time-lapse of 26 hours PF trunk vasculature of *MO-wasb* Tg[(fli1ep:Lifeact-EGFP); Tg(fli1:NLS-mCherry)] embryo. Time frame 10 min. White arrowhead (min 0) - anastomosis. Black arrow head (min 180) - secondary sprouting. Yellow circle (min 430, 490) - mitosis (strong cortical F-actin accumulation). Blue arrowhead (min 530) - emigration to the DLAV. Red outline arrowhead (min 1110) - emigration to DLAV.

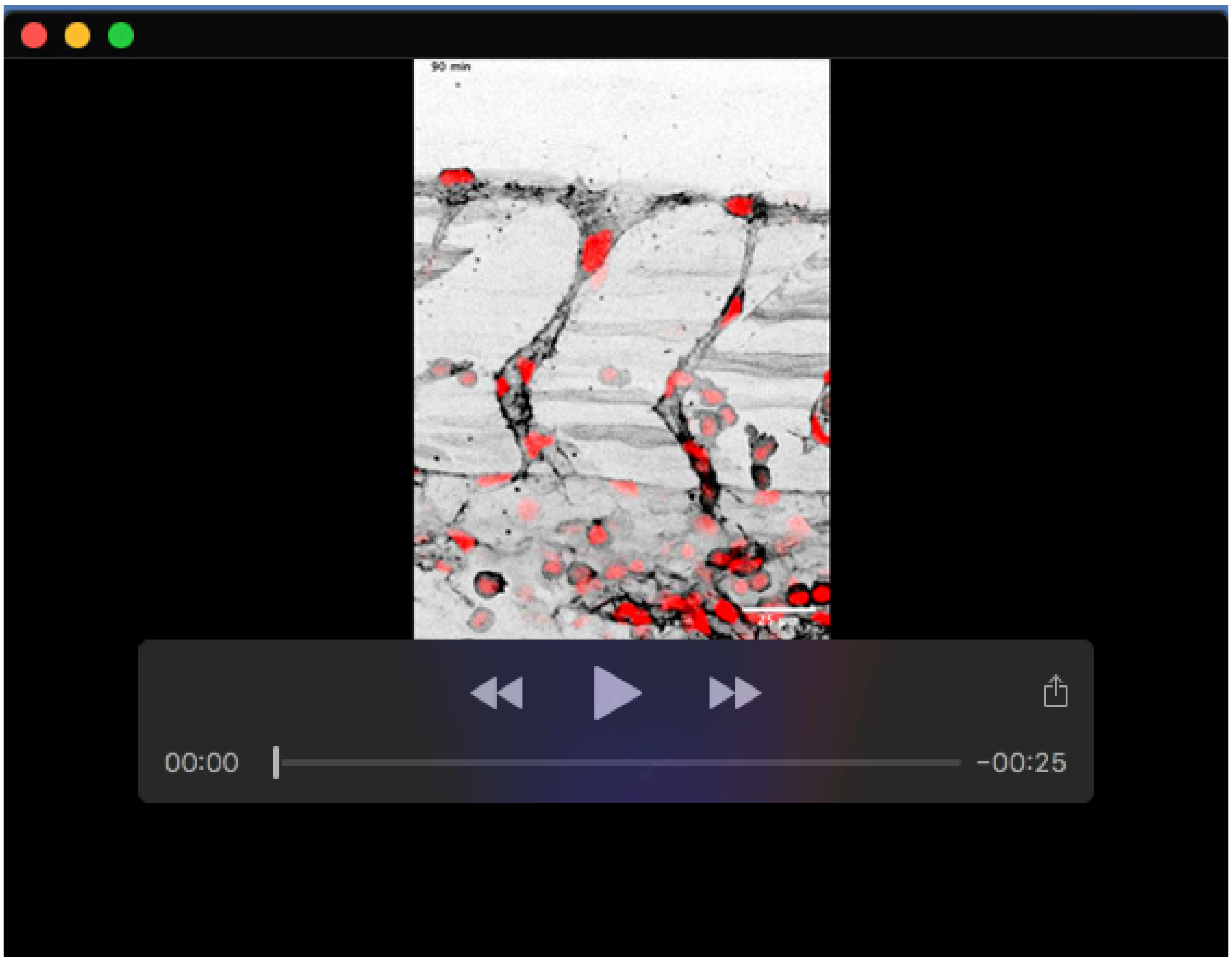

Movie 5. Time-lapse of 26 hours PF trunk vasculature of *MO-gata1* Tg[(fli1ep:Lifeact-EGFP); Tg(fli1:NLS-mCherry)] embryo. Time frame 10 min. White arrowhead (min 0)- anastomosis. Black arrow head (min 540) - secondary sprouting. Yellow circle (min 1030) - mitosis (strong cortical F-actin accumulation). Blue arrowhead (min 340) - emigration to the DLAV. Red arrowhead (min 620) - emigration to DLAV.

Development • Supplementary information

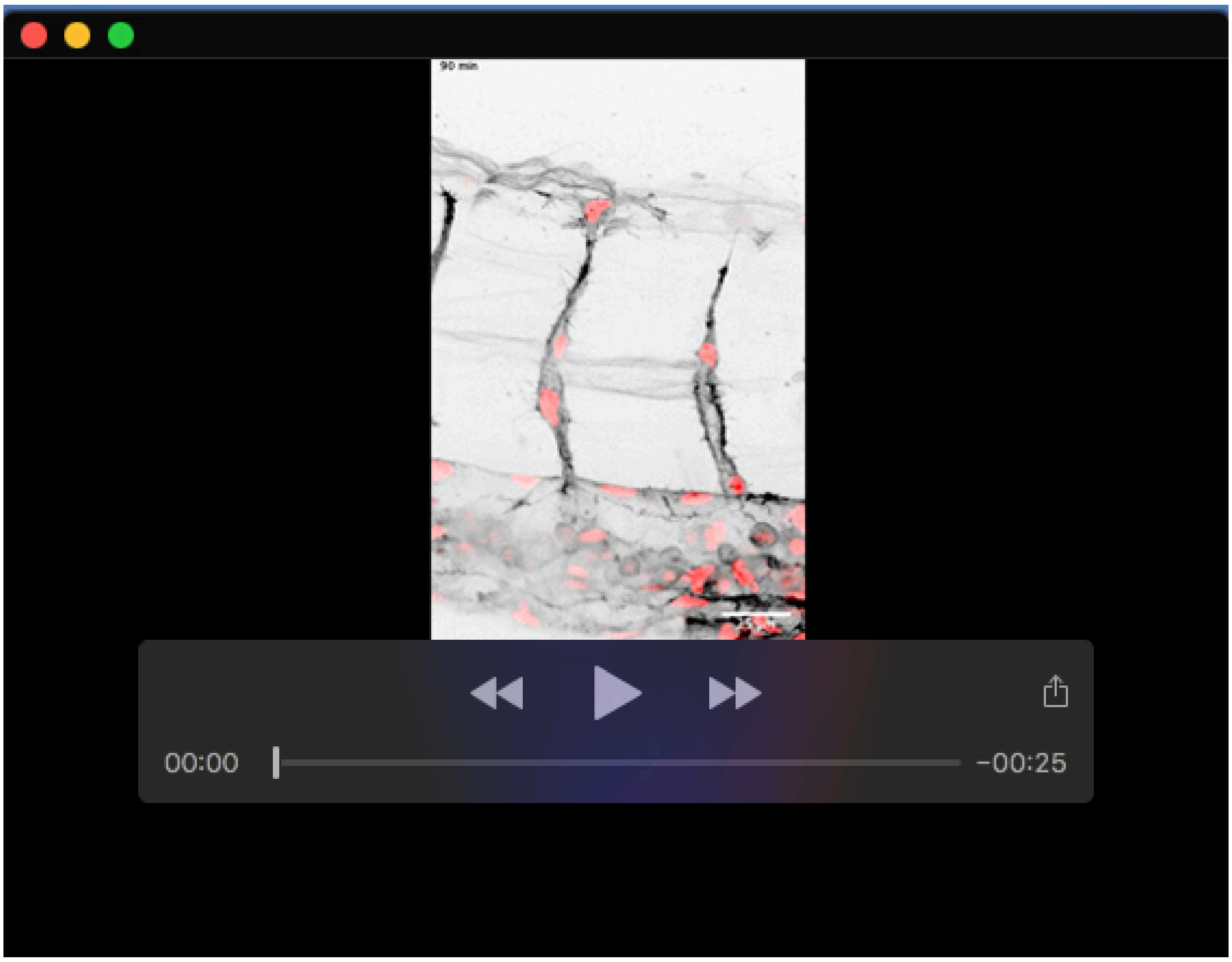

Movie 6. Time-lapse of 26 hours PF trunk vasculature of *MO-gata1/wasb* Tg[(fli1ep:Lifeact-EGFP); Tg(fli1:NLS-mCherry)] embryo. Time frame 10 min. White arrowhead (min 180) - anastomosis. Black arrow head (min 290) - secondary sprouting. Yellow circle (min 230, 550) - mitosis (strong cortical F-actin accumulation). Blue arrowhead (min 360) - emigration to the DLAV.
